# Prognostic value of LPAR1 expression and methylation in low-grade gliomas: a meta-analysis of TCGA and CGGA datasets and functional validation

**DOI:** 10.1186/s12885-025-15406-z

**Published:** 2025-12-30

**Authors:** Hongmin Chen, Wenyi Liang, Zhang Li, Chenbin Bian, Hongbin Wang, Song Luo, You Xin, Wang Feng, Liang Liang

**Affiliations:** 1 Cancer Center, Sichuan Provincial People’s Hospital, University of Electronic Science and Technology of China, Chengdu, 610072 China; 2 Sichuan University, Chengdu, Sichuan 610041 China; 3 Department of Medical Oncology, Cancer Center, West China Hospital, West China Medical School, Sichuan University, Sichuan, China; 4 Yuechi Hospital of Traditional Chinese Medicine, Guangan, Sichuan 638300 China; 5 Department of Abdominal Oncology, Cancer Center, West China Hospital, West China Medical School, Sichuan University, Sichuan, China; 6 Chinese Academy of Sciences Sichuan Translational Medicine Research Hospital, Chengdu, 610072 China

**Keywords:** Lysophosphatidic acid receptor 1 (LPAR1), Low-grade gliomas (LGG), Methylation, Prognosis, Immune infiltration infiltrating cells (TIICs)

## Abstract

**Background:**

Lysophosphatidic acid receptor 1 (LPAR1) mediates various biological behaviors in physiological and pathological processes. This study aims to comprehensively evaluate the prognostic value of LPAR1 expression and methylation in LGG, and explore their functional effects on tumor progression and immune regulation.

**Methods:**

The GEO database was used to analyze LPAR1 expression in tumors and normal tissues. The TCGA-LGG and CGGA datasets were used to analyze the expression, methylation, immunity and prognostic significance of LGG. Immune cells and immune pathways were detected by flow cytometry, ELISA and western blot analysis. The role of LPAR1 in LGG was validated by RT-PCR, TUNEL assay, CCK-8 assay, flow cytometry, wound healing assay and transwell assay.

**Results:**

We included 627 patients who contained complete information required for analysis in the TCGA-LGG and CGGA datasets. Methylation of the LPAR1 promoter suppresses its expression. High methylation levels were associated with better overall survival (OS) and progression-free survival (PFS) (*P* < 0.05) in LGG. Decreased LPAR1 expression and increased methylation levels were significantly associated with age, histological types and isocitrate dehydrogenase (IDH) mutation status (*P* < 0.05). Multivariate analysis showed that LPAR1 was an independent prognostic factor for LGG (*P* = 0.002). Meta-analysis showed that high LPAR1 expression was indeed a poor prognosis for patients’ OS in LGG (HR, 1.06; 95% CI, 0.95–1.17). Low LPAR1 expression affected the proportion of tumor immune infiltrating cells (TIICs) in the tumor microenvironment (TME), which was related to the involvement of LPAR1 in pathways such as CSK and Th1/Th2. In the cell experiment, LPAR1 overexpression promoted proliferation, migration, and invasion and inhibited apoptosis, whereas LPAR1 knockdown exhibited the opposite effect.

**Conclusions:**

Low LPAR1 expression or high LPAR1 methylation level was a potential prognostic molecular marker for favorable survival in LGG. Moreover, we identified a regulatory pattern in which a high level of LPAR1 methylation resulted in a decrease in LPAR1 expression, which affected the proportion of TIICs in TME. This pattern may work through CSK and Th1/Th2 pathways. Inhibiting LPAR1 can suppress the biological behavior of glioma cells in vitro. These findings open up new avenues for therapeutic and prognostic evaluation of LGG.

**Supplementary Information:**

The online version contains supplementary material available at 10.1186/s12885-025-15406-z.

## Introduction

Low-grade glioma (LGG) is generally defined as primary central nervous system tumor classified by the World Health Organization (WHO) as grade 2 and grade 3 with isocitrate dehydrogenase (IDH) mutations without GBM molecular events [1]. They account for 10–20% of all primary brain tumors, which are usually in an inactive state. The type of gliomas differs not only histologically but also in clinical presentation. WHO grade 1 tumor usually occur in children and are considered benign; however, a few cases with anaplastic features or molecular variants, such as MAP2K1 mutation and CDKN2A/B homozygous deletion, may be upgraded to a higher grade, increasing the risk of recurrence [2]. Grade 2 and 3 are predominantly astrocytoma and oligodendroglioma, which are accompanied by IDH mutations and represent the majority of LGGs. while glioblastoma, IDH-wildtype is classified as a grade 4, which is most malignant with the presence of altered genetic parameters and pathological features [1, 3]. The proliferation and progression of LGG vary greatly. The median survival time of astrocytoma is about 10 to 11 years, and that of oligodendroglioma is 15 years [4]. Traditional factors are still believed to affect the final prognosis, including age (40 or older), histological subtype, preoperative maximum tumor diameter, neurological deficits [5]. Clinicians are increasingly concerned about genetic changes in clinical decision-making. One of the typical example of these was isocitrate dehydrogenase 1 (IDH1) mutations, which are the main feature of adult low-grade gliomas, and a good prognostic marker [6]. Furthermore, the revised classification of CNS tumors published by WHO in 2021 combines histopathological and molecular features to define these tumor entities. The molecular phenotype trumps the histopathological one and depends primarily on the IDH enzyme mutation status in this classification [7]. Despite intensive therapies, including surgery, radiotherapy, and chemotherapy, the outcome of glioma patients remains dismal [8–10]. Therefore, the identification of novel molecular markers is of great significance for LGG treatment and prognosis.

Lysophosphatidic acid (LPA, 1-acyl-2-lyso-sn-glycerol-3-phosphate), a small glycerophosphatidic acid, widely exists in human body [11, 12]. LPA combines with a variety of known G-protein coupled receptors (GPCRs) to perform a wide range of biological functions [13]. Lysophosphatidic acid receptor 1 (LPAR1) is one of the G protein-coupled receptors. It participates in a variety of biological functions by binding to lysophosphatidic acid (LPA), including chemotaxis, proliferation, cell differentiation, platelet aggregation and tumor progression [14]. Recent reports indicate that LPAR1 is a prognostic marker for a variety of tumors and plays an important role in the occurrence and development of gastric cancer, prostate cancer, and ovarian serous cystadenocarcinoma [15–17]. Moreover, there is evidence that transmembrane proteins can exacerbate the malignant phenotype of gliomas through the classical Wnt /β connexin signaling pathway [18]. However, the role of LPAR1 in LGG tumor progression has not been reported.

Therefore, this study will reveal the research value of LPAR1 expression and methylation in low-grade gliomas based on datasets from multiple data sources. Then, the role of LPAR1 in gliomas will be validated in vitro by knocking down or overexpressing LPAR1, and possible mechanisms were explored.

## Materials and methods

### Data acquisition

This study included patients who were first diagnosed and underwent surgical treatment between January 2005 and December 2015. The inclusion criteria were histological diagnosis of World Health Organization (WHO) grade 2 or 3 astrocytoma and oligodendroglioma (LGG). Complete whole genome expression data (RNA Seq), DNA methylation data, and detailed clinical pathological information (such as age, IDH mutation status, 1p/19q co deletion status, follow-up time, and survival status) must be obtained simultaneously. Exclude patients with incomplete clinical information, follow-up time less than 6 months, or sample quality issues. Based on the above criteria, we selected 801 patients (TCGA:510 ; CGGA: 291) from the initial samples of TCGA-LGG (https://portal.gdc.cancer.gov/projects/TCGA-LGG) [19] and CGGA (https://www.cgga.org.cn/download.jsp) [20] databases to form the final analysis cohort (Supplementary Table 1 for detailed information). In addition, both the TCGA-LGG and CGGA datasets contain some wild-type IDH patients (TCGA: 87 cases; CGGA: 87 cases), whereas wild-type IDH is not included in astrocytoma and oligodendroglioma according to the 2021 WHO guideline [7]. Therefore, as shown in Figure S1, we screened the data based on previous publications [21] and selected 627 cases (TCGA-LGG: 423 cases; CGGA: 204 cases) as the final cohort for analysis. TCGA-LGG is used to analyze the expression profile data, DNA methylation data, and corresponding clinical information of target genes. CGGA database is used as the validation set. GSE16011 (https://www.ncbi.nlm.nih.gov/geo/query/acc.cgi?acc=GSE16011) [22] contains expression profile data for 8 normal tissues, 32 low-grade gliomas, and 244 high-grade gliomas (HGG). We used the GEO dataset to investigate the expression information of target genes in normal tissues and different grades of gliomas.

### Data preprocessing

Download GEO16011 data from the GEO database, preprocess missing values using R language data and platform probes, normalize them, annotate probes and map genes, and finally correct batch effects using ComBat. Download TCGA-LGG count data from the TCGA database, clean and integrate the data in R language, and standardize and correct batch effects using DESeq. Download TCGA-LGG methylation data from the TCGA database, standardize using BMIQ, and correct batch effects using ComBat. Download data from CGGA database and use R language for data cleaning and integration.

### Kaplan Meier survival analysis and TIMER database analysis

The overall survival (OS) and progression free survival (PFS) were visualized using Kaplan Meier curves, and the differences between the two groups were compared using Log rank test. Use Cox proportional hazards model to adjust for confounding factors such as age and IDH status. Analysis of the abundance of tumor infiltrating immune cells (TIIC) and its correlation with target gene expression using TIMER 2.0 web server (http://timer.cistrome.org/). This analysis was conducted in the Cancer Genome Atlas Low Grade Gliomas (TCGA-LGG) cohort, which included 423 patient samples. The correlation between target gene expression and immune infiltration level calculated by TIMER 2.0 is expressed as a partial correlation value adjusted for tumor purity. If the p-value is less than 0.001 and the partial correlation coefficient exceeds 0.25, the result is considered statistically significant.

### Differential analysis and enrichment analysis

We divided LGG patients into a high expression group and a low expression group (based on the median LPAR1 expression), used the Wilcox.test function in R language, and performed multiple comparisons and adjustments of p-values using the Benjamini Hochberg method to control for false discovery rate (FDR). The genes with adjusted p (FDR) < 0.05 and |(log2FC)|>1 are considered to have statistically significant differential expression. The annotation function of gene ontology (GO) analysis is comprised of three categories: biological process (BP), cellular component (CC), and molecular function (MF). GO analysis of candidate DEGs were performed using the R package “clusterProfiler”. Adjusted p-value less than 0.05 was considered as the cut-off criterion. Gene set enrichment analysis (GSEA) is a computational method that determines whether a priori defined set of genes shows statistically significant and concordant differences between two biological states [23]. Significant enrichment criteria: the absolute value of normalized enrichment score (NES) is greater than 1, the nom pvalue is less than 0.05, and the FDR qvalue is less than 0.25. The gene set variation analysis (GSVA) R package was employed to verify the biological processes in the two groups stratified as described above [24].

### Meta-analysis and model comparison and improvement assessment

A meta-analysis of two large independent cohorts, TCGA-LGG and CGGA, was conducted to evaluate the prognostic value of LPAR1 expression on overall survival in patients with low-grade glioma (LGG). The hazard ratio (HR) and its 95% confidence interval (CI) were used as the effect size measure for each cohort. Statistical heterogeneity between the two datasets was assessed using Cochran’s Q test and quantified with the I² statistic. A fixed-effect model was applied if I² < 50%; otherwise, a random-effects model was used. All meta-analyses were performed using the ‘meta’ package in R, with statistical significance set at *P* < 0.05.

To evaluate the net improvement in prognostic performance contributed by LPAR1, we constructed two sets of Cox proportional hazards models for overall survival (OS). The baseline multivariable model included established clinical prognostic factors such as age, histological grade, and IDH mutation status. The LPAR1-only model contained LPAR1 expression as the sole variable. The integrated model combined all variables from the baseline model with LPAR1 expression. The prognostic performance of these models was compared using the Concordance Index (C-index) and its 95% confidence interval, calculated via bootstrap resampling (1000 repetitions). The difference in C-index between the integrated model and the baseline model was tested for statistical significance.

### Cell culture and transfection

Human neuroglial cells (SNP-H236) and four representative glioblastoma multiforme (GBM) cell lines, namely A172, LNZ-308, U251, and U87 are purchased from Thermo Fisher Scientific. SW1088 cells was purchased from the American Type Culture Collection (ATCC, USA). These cells were grown in DMEM supplemented with 10% fetal bovine serum (FBS) and 1% penicillin-streptomycin solution. Regarding cell transfection, cells were transfected with siRNA (Genomeditec, Shanghai, China) using Lipofectamine 3000 (Thermo Fisher Scientific, USA).

### Cell viability assay (CCK-8)

Cell viability was assessed using the Cell Counting Kit-8 (CCK-8; Dojindo, Japan) according to the manufacturer’s instructions. In brief, cells were seeded in 96-well plates at a density of 5 × 10^3^ cells per well and incubated for 24 h. Following the respective treatments, 10 µL of CCK-8 reagent was added to each well, and the plates were incubated at 37 °C for an additional 2 h. The absorbance of each well was then measured at a wavelength of 450 nm using a microplate reader (BioTek, USA). Viability was calculated as a percentage relative to the untreated control group.

### TUNEL staining assay

Apoptotic cells were detected using the Terminal deoxynucleotidyl transferase dUTP Nick-End Labeling (TUNEL) assay kit (Roche, Switzerland). Briefly, cells grown on coverslips were fixed with 4% paraformaldehyde for 30 min at room temperature and permeabilized with 0.1% Triton X-100 for 5 min on ice. After washing with PBS, the cells were incubated with the TUNEL reaction mixture for 1 h at 37 °C in the dark. Apoptotic cells (TUNEL-positive) were visualized and counted using a fluorescence microscope (Nikon, Japan).

### Wound healing assay

Cell migration was evaluated using a wound healing assay. Cells were cultured in 6-well plates until they reached 100% confluence. A sterile 200 µL pipette tip was used to create a scratch wound across the cell monolayer. The detached cells were removed by washing with PBS. Fresh serum-free medium was added, and the wounded areas were photographed at 0 and 24 h under an inverted microscope (Olympus, Japan). The migration distance was quantified using ImageJ software (NIH, USA).

### Transwell assay

Cell invasion was assessed using 24-well Transwell chambers (Corning, USA) coated with Matrigel (BD Biosciences, USA). Briefly, 5 × 10^4^ cells suspended in 200 µL of serum-free medium were plated into the upper chamber. The lower chamber was filled with 500 µL of complete medium containing 10% FBS as a chemoattractant. After 24 h of incubation, non-invading cells on the upper surface of the membrane were gently removed with a cotton swab. The invaded cells on the lower surface were fixed with methanol, stained with 0.1% crystal violet, and counted from five random fields under a microscope.

### Flow cytometry

For apoptosis analysis, cells were stained with Annexin V-FITC and PI using an apoptosis detection kit (BD Biosciences, USA) according to the protocol. The stained cells were analyzed using a flow cytometer (BD FACS-Canto II, USA), and the data were processed with FlowJo software (Tree Star, USA).

Single-cell suspensions were prepared from spleen or peripheral blood. Cells were incubated with anti-CD16/32 antibody to block Fc receptors. Surface staining was performed using fluorochrome-conjugated antibodies against CD3, CD4, and CD19 for 30 min at 4℃ in the dark. Cells were washed and analyzed on a flow cytometer. Lymphocytes were gated based on FSC/SSC properties, followed by singlet gating. CD4 + T cells were identified as CD3+/CD4 + cells, and B cells as CD19 + cells. Their ratio was calculated from the frequencies within the lymphocyte gate using FlowJo software.

### In vitro methylation assay

Methylation of synthetic oligonucleotides or plasmids was performed using the CpG Methyltransferase (M.SssI; New England Biolabs, USA) according to the manufacturer’s instructions. The reaction mixture was incubated at 37 °C for 4 h, followed by enzyme heat inactivation at 65 °C for 20 min. The methylated DNA was purified using a PCR purification kit (Qiagen, Germany) and verified by digestion with the methylation-sensitive restriction enzyme HpaII.

### Dual-luciferase reporter assay

The fragments of LPAR1 promoter that contained cg14133557 site were cloned into the luciferase reporter vector pmirGLO (Promega, USA). Cells were transfected with the above reporter construct using Lipofectamine 3000 (Invitrogen, USA) then treated with CpG methylase M. ssI. After 48 h, Firefly luciferase and Renilla luciferase activity levels activities were measured sequentially using the Dual-Luciferase Reporter Assay System (Promega, USA) on a GloMax luminometer (Promega, USA). Firefly luciferase activity was normalized to Renilla luciferase activity for each sample.

### Enzyme-Linked Immunosorbnent Assay (ELISA)

The concentrations of CD19 (whole-cell lysates), TNF-α and IL-4 (cell-culture supernatants) were quantified using specific commercial ELISA kits (R&D Systems, USA) following the provided protocol. Standards and samples were added to antibody-precoated 96-well plates and incubated. After a series of washes and incubation with a conjugated detection antibody, the substrate solution was added. The reaction was stopped, and the absorbance was measured at 450 nm. Concentrations were determined from a standard curve.

### Quantitative Real-Time PCR (RT-qPCR)

Total RNA was extracted from cells using TRIzol reagent (Invitrogen, USA). cDNA was synthesized from 1 µg of RNA using a PrimeScript RT reagent kit (Takara, Japan). Quantitative PCR was performed on a QuantStudio 5 Real-Time PCR System (Applied Biosystems, USA) using SYBR Green Premix (Takara, Japan). The relative mRNA expression levels were calculated using the 2^(-ΔΔCt) method and normalized to the expression of GAPDH. All primers were designed to span intron-exon junctions.

### Western blot analysis

Total protein was extracted from cells using RIPA lysis buffer containing protease and phosphatase inhibitors. Protein concentration was determined with a BCA assay kit (Thermo Fisher Scientific, USA). Equal amounts of protein were separated by SDS-PAGE and transferred onto PVDF membranes. The membranes were blocked with 5% non-fat milk and then incubated with primary antibodies overnight at 4 °C, followed by incubation with HRP-conjugated secondary antibodies. Antibodies LPAR1, CSK, LCK, CD25, T-bet, IFN-γ, GATA3, IL-4 and GAPDH were purchased from Proteintech. Antibody p-LCK was from ABclonal. Protein bands were visualized using an ECL detection system (Bio-Rad, USA) and imaged with a chemiluminescence imaging system. GAPDH served as the loading control.

### Statistical analysis

All statistical analyses were conducted using R (v.3.6.2). The relationship between clinicopathologic characteristics and LPAR1 expression or methylation was analyzed with the Chi-square test. Univariate and multivariate Cox regression models was used to study the correlation between LPAR1 and OS in LGG patients. Pearson correlation coefficient was used to detect the correlation between LPAR1 expression and methylation level. Kaplan-Meier curve was used to compare the survival difference between high and low LPAR1 expression and methylation levels. The cut-off value of LPAR1 expression and methylation was determined by its median value. Statistical difference in experimental data was analyzed by unpaired Student’s *t*-test or one-way analysis of variance (ANOVA). All of data are presented as the mean ± SD *P* < 0.05 manifested statistical significance.

## Result

### High expression and low promoter methylation of LPAR1 correlate with poor prognosis

**Fig. 1:**
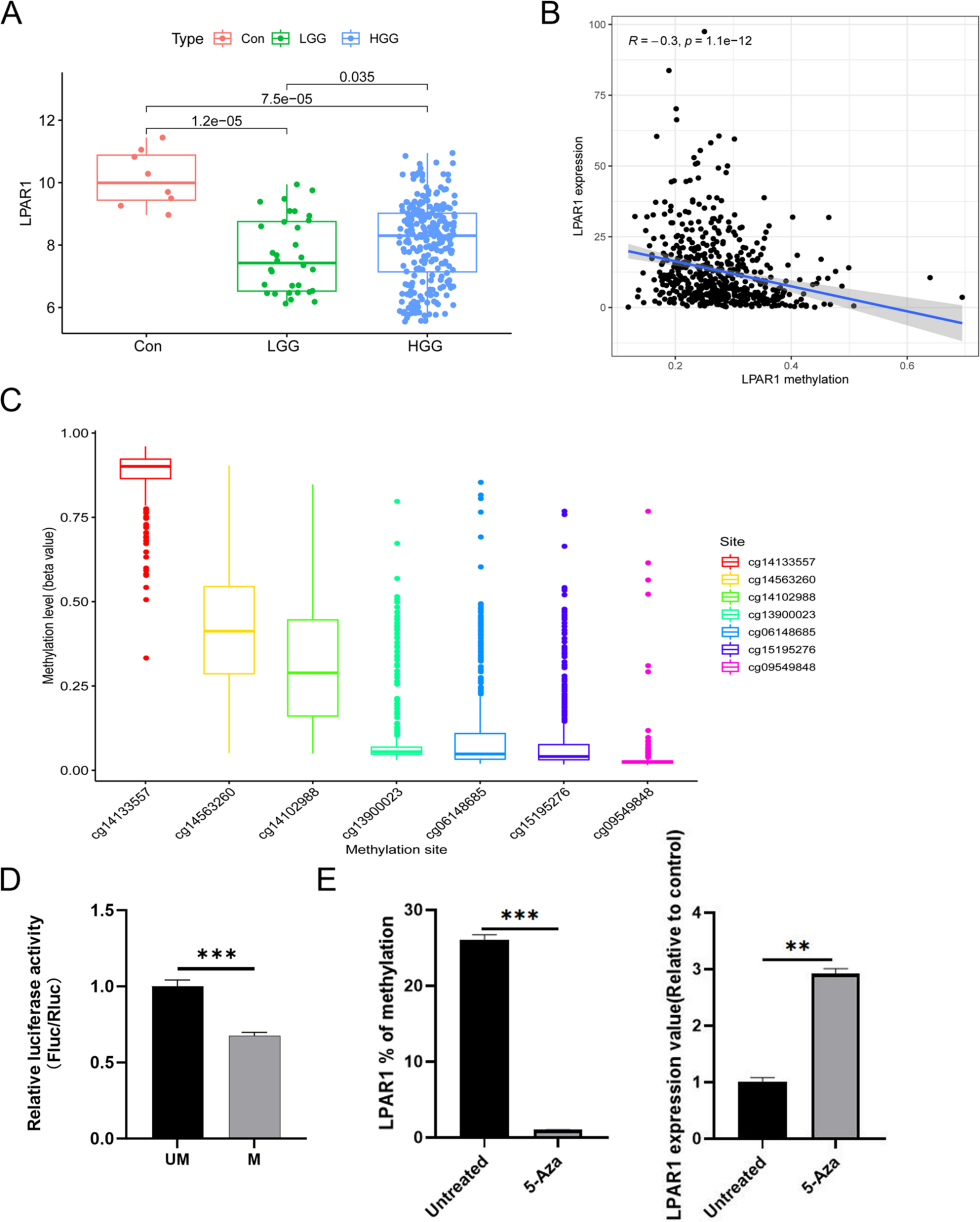
LPAR1 expression is negatively correlated with its promoter methylation. **A** Increased expression of LPAR1 in glioma progression; **B** negative correlation between the expression of LPAR1 and its methylation level; **C** expression levels of seven methylation sites in the promoter region of the LPAR1 gene. **D** The cg14133557 site of the LPAR1 promoter was verified in vitro using CpG methylase M. ssI. **E** The effect of DNA methylation-specific inhibitor 5-Aza on LPAR1 methylation and gene expression. Student’s *t*-test was employed for (**D** and **E**). Data are presented as mean ± SD, *P < 0.05, ** *P* < 0.01, *** *P* < 0.001

We first compared the expression of LPAR1 in gliomas and found that LPAR1 expression was downregulated in overall gliomas compared with normal tissues; however, LPAR1 expression was significantly higher in high-grade gliomas than in LGG (Fig. 1A). LPAR1 expression was negatively correlated with its promoter methylation level (Fig. 1B; *R* = -0.3; *P* < 0.001). Figure 1C clearly revealed the methylation sites and expression level of LPAR1 promoter. Pearson correlation analysis showed that LPAR1 was negatively correlated with the methylation levels of cg14102988, cg14133557, cg15195276, cg13900023, cg06148685 and cg14563260 (*R* < − 0.1; *P* < 0.001), but not with cg09549848 (Figure S2). We used Kaplan-Meier survival curve to explore the effect of LPAR1 and its methylation level on the survival of patients. Low expression of LPAR1 not only represents a good OS in LGG patients (Figure S3A; *P* < 0.001), but also a favorable PFS (Figure S3B; *P* = 0.0057). These have also been confirmed in the CGGA dataset (Figure S3C; *P* < 0.001). We further performed a stratification analysis to investigate the prognostic value of LPAR1 expression. The patients were grouped according to age (≤ 45 and > 45), gender (female and male), histological grade (G2 and G3), histological types (astrocytoma and oligodendroglioma). As shown in Figure S4A-H, the OS was significantly shorter for the high-LPAR1 patients compared to the low-LPAR1 patients among ages ≤ 45 (*P* = 4.613e-02), age > 45 (*P* = 4.092e-02), male patients (*P* = 2.662e-02), female patients (*P* = 9.303e − 03), G2 (*P* = 3.073e-02), G3 (*P* = 3.746e-03) and astrocytoma (*P* = 4.472e-04). However, the OS of oligodendroglioma (*P* = 8.89e-01) were similar in LGG patients between high-LPAR1 and low-LPAR1. Kaplan Meier survival curve displayed that most methylation sites were associated with OS or PFS in LGG patients, especially cg06148685, cg14133557, cg15195276 (Figure S5A-B; all *P* < 0.05). Next, to verify the effect of methylation on LPAR1 expression, we selected cg14133557 in the LPAR1 promoter for in vitro methylation experiments, which reduced the fluorescence intensity after adding methylase in M group (Fig. 1D). Furthermore, we found that inhibition of methylation did promote LPAR1 expression (Fig. 1E).

### LPAR1 expression and methylation are associated with multiple clinicopathological characteristic

**Fig. 2:**
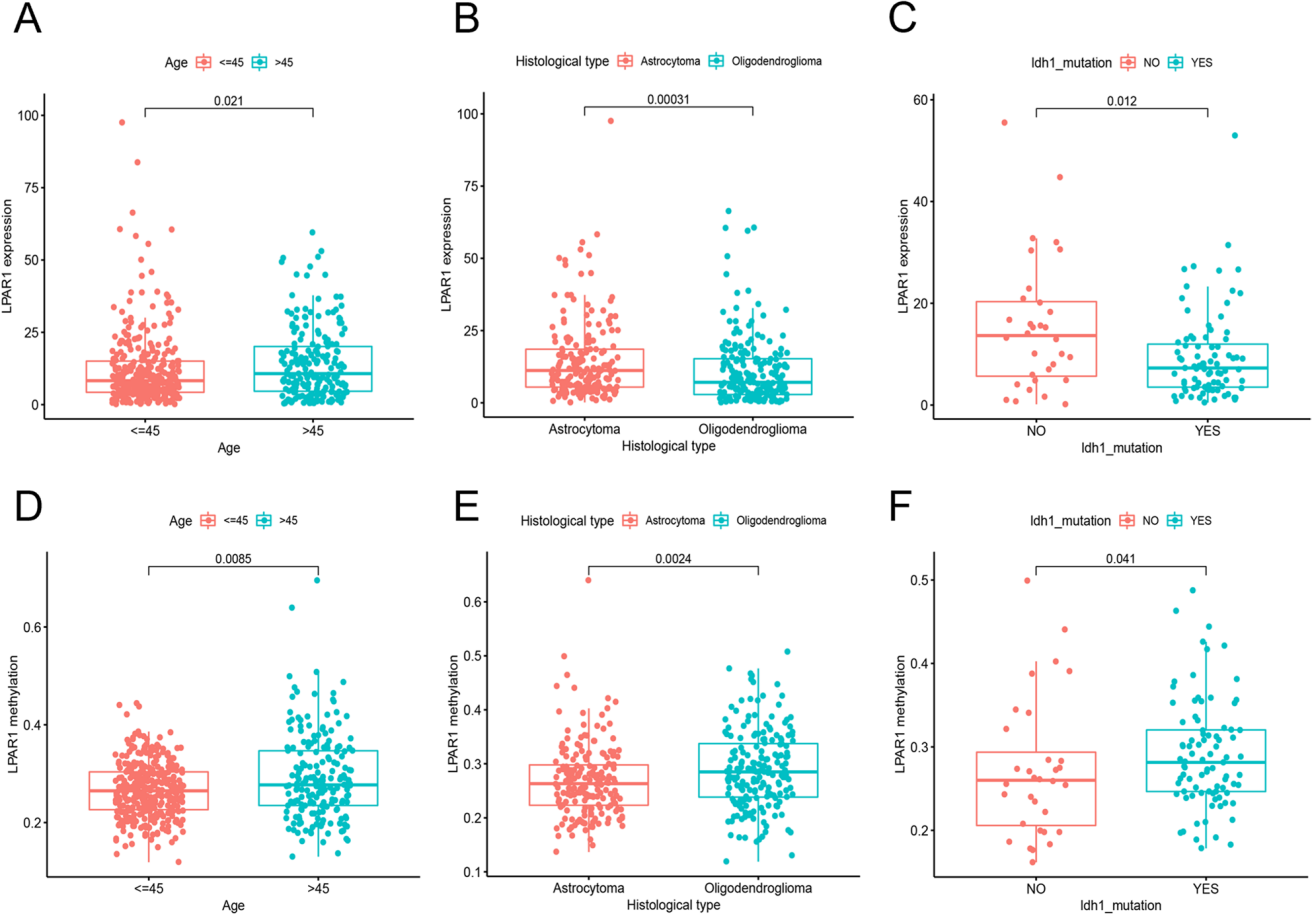
Correlation analysis showed that LPAR1 expression or methylation was associated with various clinicopathological features of LGG patients. The analysis compared the expression of LPAR1 in LGG patients according to (**A**) Age; **B** Histological types; **C** IDH mutation status. The analysis was based on age (**D**); **E** histological type; **F** IDH mutation status to compare the methylation level of LPAR1 in LGG patients

Analyze all patient characteristic data collected from 423 TCGA-LGG sample (Table 1). As shown in Fig. 2A and B, LPAR1 is highly expressed in the elderly group (age > 45 years; *P* = 0.021) and the astrocytoma group (*P* = 0.00031). In addition, we noticed a significant increase in LPAR1 expression in the IDH wild-type group of the TCGA-LGG database. The analysis results of the CGGA dataset show consistent conclusions: LPAR1 is highly expressed in astrocytomas (Figure S6A; *P* < 0.05). Meanwhile, LPAR1 expression also significantly increased in the IDH wild-type group in the CGGA database (Figure S6B). It is worth noting that the expression of LPAR1 is higher in males than in females (Figure S6C; *P* = 0.011). The expression in the 1p19q non co deletion group was higher than that in the 1p19q co deletion group (Figure S6D; *P* < 0.001). Further explore the correlation between LPAR1 methylation status and clinical pathological features through chi square test. The methylation level of LPAR1 was higher in the elderly group and the astrocytoma group, and showed a further increasing trend in the IDH wild-type group (Fig. 2D-F).

**Table 1:** The relationship between LPAR1 expression, methylation and clinical features

Clinical Characteristics		LPAR1 expression			LPAR1 methylation		
		High %	Low %	*P* value	High %	Low %	*P* value
Age	<=45	145(61.7%)	112(59.57%)	0.016	117(57.07%)	132(60.83%)	0.0409
	> 45	90(38.3%)	76(40.43%)		89(42.93%)	85(39.17%)	
Histologic grade	G2	100(45.87%)	105(51.22%)	0.5006	100(49.02%)	93(42.86%)	0.4075
	G3	118(54.13%)	100(48.78%)		105(50.98%)	124(57.14%)	
Tumor location	Frontal Lobe	120(53.81%)	103(51.76%)	0.7266	112(54.63%)	111(51.15%)	0.5424
	Parietal Lobe	25(10.76%)	24(12.06%)		24(11.22%)	25(11.52%)	
	Temporal Lobe	78(34.98%)	70(35.18%)		69(33.66%)	79(36.41%)	
	unknown	1(0.45%)	2(1.01%)		1(0.49%)	2(0.92%)	
Gender	Female	100(43.29%)	85(44.27%)	0.8271	90(41.86%)	121(55.76%)	0.3954
	Male	131(56.71%)	107(55.73%)		115(58.14%)	96(44.24%)	
Cancer status	Tumor free	80(34.48%)	58(29.15%)	0.8566	68(33.17%)	76(35.02%)	0.4084
	With tumor	99(42.67%)	90(45.23%)		89(43.41%)	86(39.17%)	
	unknown	53(22.84%)	51(25.63%)		48(23.41%)	56(25.81%)	
Histological type	Astrocytoma	100(39.22%)	91(37.14%)	0.031	65(30.66%)	83(38.25%)	0.014
	Oligodendroglioma	90(35.29%)	90(36.73%)		90(42.45%)	63(28.57%)	
	unknown	65(25.49%)	64(26.12%)		57(26.89%)	72(33.18%)	
LPAR1 expression	High	—	—	—	100(48.78%)	116(53.46%)	< 0.001
	Low	—	—		106(51.22%)	101(46.54%)	
LPAR1 methylation	High	100(44.64%)	134(67.34%)	< 0.001	—	—	—
	Low	124(55.36%)	65(32.66%)		—	—	
IDH1 mutation	YES	31(12.2%)	56(21.96%)	0.0066	49(19.29%)	38(14.9%)	0.1892
	NO	21(8.27%)	11(4.31%)		13(5.12%)	19(7.45%)	
	unknown	202(79.53%)	189(73.73%)		192(75.59%)	199(77.65%)	

*IDH1* Isocitrate dehydrogenase 1, *LPAR1* Lysophosphatidic acid receptor 1

### LPAR1 expression is an independent prognostic factor for LGG

**Fig. 3:**
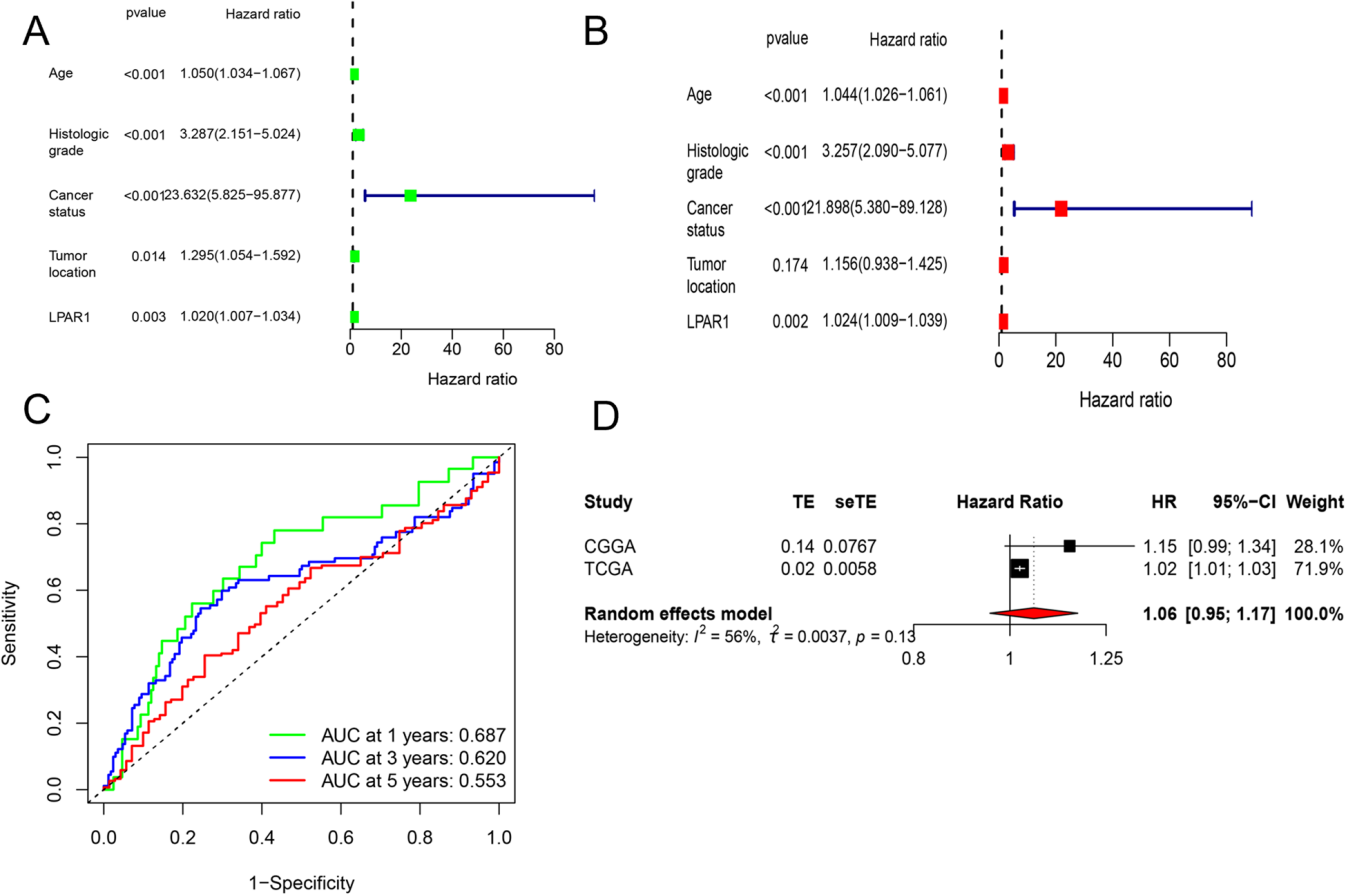
Estimation of the prognostic accuracy of the LPAR1 expression in the LGG patients. **A** Univariate Cox regression analysis of the correlation between OS and various clinicopathological parameters and LPAR1; **B** Multivariate Cox regression analysis; **C** Receiver operating characteristic curve analysis shows the prognostic accuracy of LPAR1 expression; **D** Comparison of Model Predictive Performance (C-index); **E** A meta-analysis of the correlation between LPAR1 expression and OS in LGG patients

We performed univariate and multivariate Cox regression analysis to determine whether LPAR1 expression is an independent prognostic factor in LGG patients. Univariate analysis showed that age (*P* < 0.001), histological grade (*P* < 0.001), cancer status (*P* < 0.001), tumor location (*P* < 0.001) and LPAR1 (*P* < 0.001) were significantly correlated with OS (Fig. 3A). Multivariate analysis displayed age (*P* < 0.001), histological grade (*P* < 0.001), cancer status (*P* < 0.001) and LPAR1 (*P* = 0.002) were independent prognostic factors for OS in LGG patients (Fig. 3B). 

The prognostic accuracy of LPAR1 was further validated using time-dependent receiver operating characteristic (ROC) analysis. The area under the curve (AUC) values for LPAR1 as a prognostic biomarker were 0.687 (95% CI: 0.624–0.750), 0.620 (95% CI: 0.556–0.684), and 0.553 (95% CI: 0.488–0.618) for 1-, 3-, and 5-year overall survival, respectively (Fig. 3C). To quantitatively assess the net improvement in prognostic performance contributed by LPAR1 beyond established clinical factors, we compared three Cox proportional hazards models (Fig. 3D). The LPAR1-only model demonstrated modest predictive ability with a C-index of 0.635 (95% CI: 0.573–0.696). The multivariable baseline model incorporating age, histological grade and IDH status showed substantially improved discrimination, achieving a C-index of 0.782 (95% CI: 0.737–0.821). Notably, the integrated model combining both clinical factors and LPAR1 expression yielded the highest predictive performance with a C-index of 0.780 (95% CI: 0.748–0.833). The addition of LPAR1 to the baseline clinical model provided a statistically significant improvement in model fit (likelihood ratio test *P* = 0.002), confirming that LPAR1 expression offers independent prognostic information beyond conventional clinical parameters.

As shown in the forest plot (Fig. 3E), a meta-analysis supported the association between high LPAR1 expression and poorer survival (pooled HR = 1.06, 95% CI: 0.95–1.17, *P* = 0.13). Crucially, integrating LPAR1 into a clinical model significantly improved prognostic performance (Likelihood ratio test *P* = 0.002), definitively establishing it as an independent unfavorable prognostic factor in LGG. 

### Low LPAR1 expression reduced the proportion of TIICs in the TME

The TIMER database was performed to retrieve the expression ratio of TIICs and the relationship between LPAR1 expression and TIICs expression. Figure S7A detailed the proportions of the B cells, CD8 + T cells, CD4 + T cells, macrophage, neutrophils and dendritic cells in LGG. The correlation analysis of TIICs showed that dendritic cells have the most positive correlation with neutrophils in LGG (Figure S7B). Using gene expression characteristics, T cell receptors and B cell receptor systems to analyze the TME to determine the immune target of neoantigens, which provides rich information for many cancer types and has prognostic value [25]. Therefore, we tried to find the relationship between the expression of LPAR1 and the level of immune infiltration in the tumor microenvironment of LGG patients. The results showed the proportion of B cells, CD4 + T cells, macrophage, neutrophils and dendritic cells were significantly reduced in low LPAR1 expression group (Figure S7C; all *P* < 0.001).

**Fig. 4:**
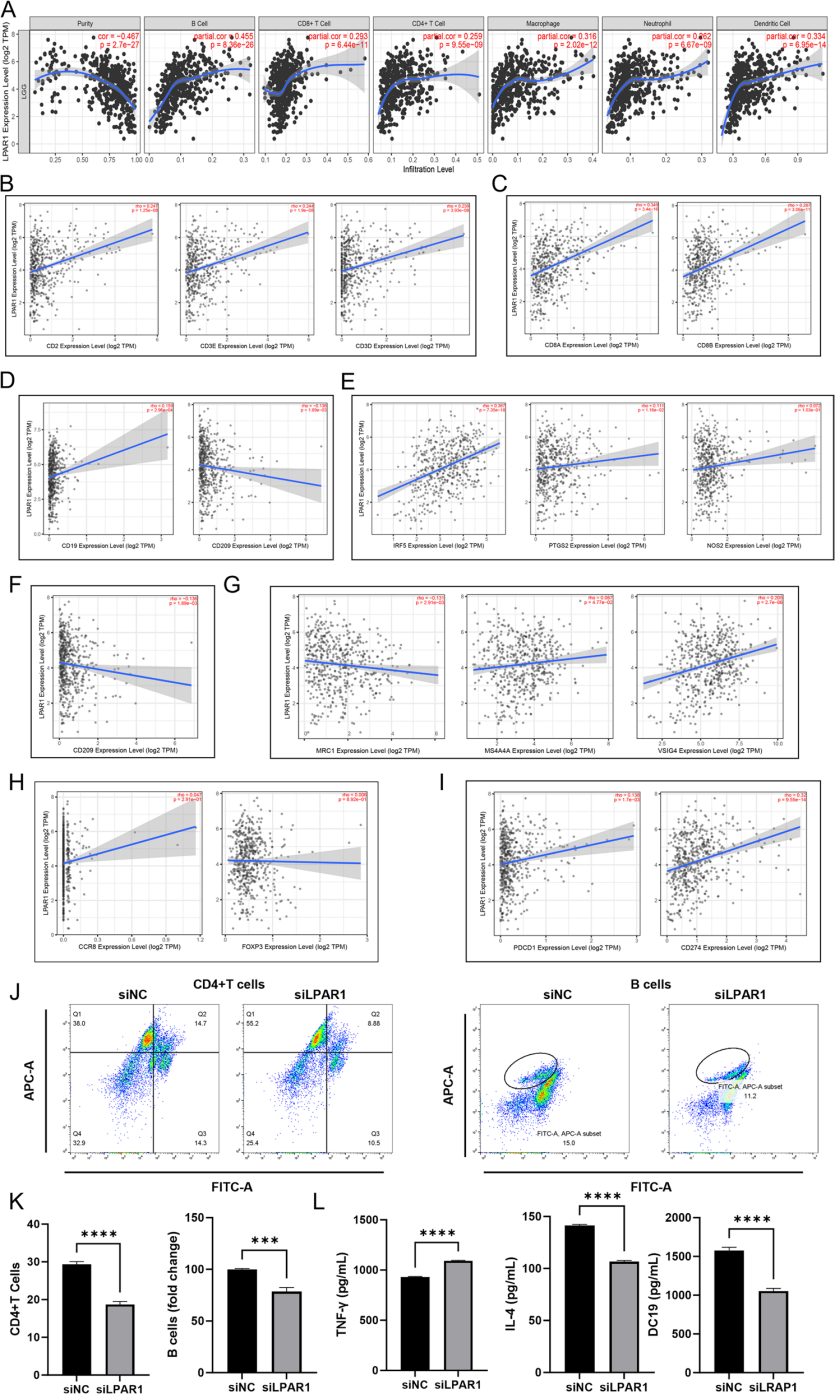
Low LPAR1 expression reduces the proportion of TIICs and their markers in TME. (A) Correlation between LPAR1 expression and infiltration of 6 kinds of immune cells. The Correlation analysis of LPAR1 expression and TIICs marker genes including (B) T cell (general); (C) CD8+ T cell; (D) B cell; (E) M1 cell; (F) dendritic cell; (G) M2 cell; (H) Tregs and (I) PD-1/PD-L1 axis. Flow cytometry to detect the number of (J) CD4+ T and B cells and (K) statistical graphs. (L) ELISA for detection of IFN-γ, IL-4 and CD19. Student’s *t*-test was employed for (J and K). Data are presented as mean ± SD, *P < 0.05, ** *P* < 0.01, *** *P* < 0.001, **** *P* < 0.0001.

Furthermore, we found that LPAR1 expression was positively correlated with 6 TIICs (Fig. 4A; all *R* >0.25; *P* < 0.0001). We further verify this result by correlation analysis. The results showed that CD2, CD3D, CD3E of T cells (general) (Fig. 4B), CD8A, CD8B of CD8 T cells (Fig. 4C), CD19, CD79A of B cells (Fig. 4D), IRF5, PTGS2 of M1 cells (Fig. 4E), CD209 of Dendritic cells (Fig. 4F) and MRC1, VSIG4 of M2 cells (Fig. 4G) had significant correlation with LPAR1 expression (|R| >0.1; *P* < 0.01). Immunotherapy is proving to be an effective therapeutic approach in a variety of cancers [26]. Regulatory T cells (Tregs) play a pivotal role in the inhibition of anti-tumor immune responses [27]. We further determined the relationship between LPAR1 expression and related markers of Tregs (Fig. 4H, I). Specially, the expression of LPAR1 was positively correlated with PD-1 (*R* = 0.13; *P* < 0.001) and PD-L1 (*R* = 0.32; *P* < 0.001).

Since the expression of LPAR1 was related to the level of immune infiltration of LGG, and the low expression of LPAR1 was related to the favorable OS of LGG. We speculate that the effect of LPAR1 expression on the prognosis of LGG patients was related to the level of tumor immune infiltration. Kaplan-Meier curve demonstrated that except B cells (Figure S8A), high expression of LPAR1 and enrichment in CD4 + T cells (HR = 2.58; *P* < 0.001), macrophages (HR = 1.69; *P* < 0.05), neutrophils (HR = 3.25; *P* < 0.001) and dendritic cells (HR = 4.26; *P* < 0.001) were associated with poorer prognosis in LGG patients (Figure S8B-F). To validate the above findings, we further validated them in SW1088, which was regarded as an in vitro model of LGG [28]. As shown in Fig. 4J and K, after co-culturing SW1088 cells with CD4 + T cells and B cells, the number of CD4 + T and B cells decreased after LPAR1 knockdown. Correspondingly, IFN-γ rose while IL-4 and CD19 was downregulated (Fig. 4L).

### LPAR1 regulates immune signaling pathways in LGG

**Fig. 5:**
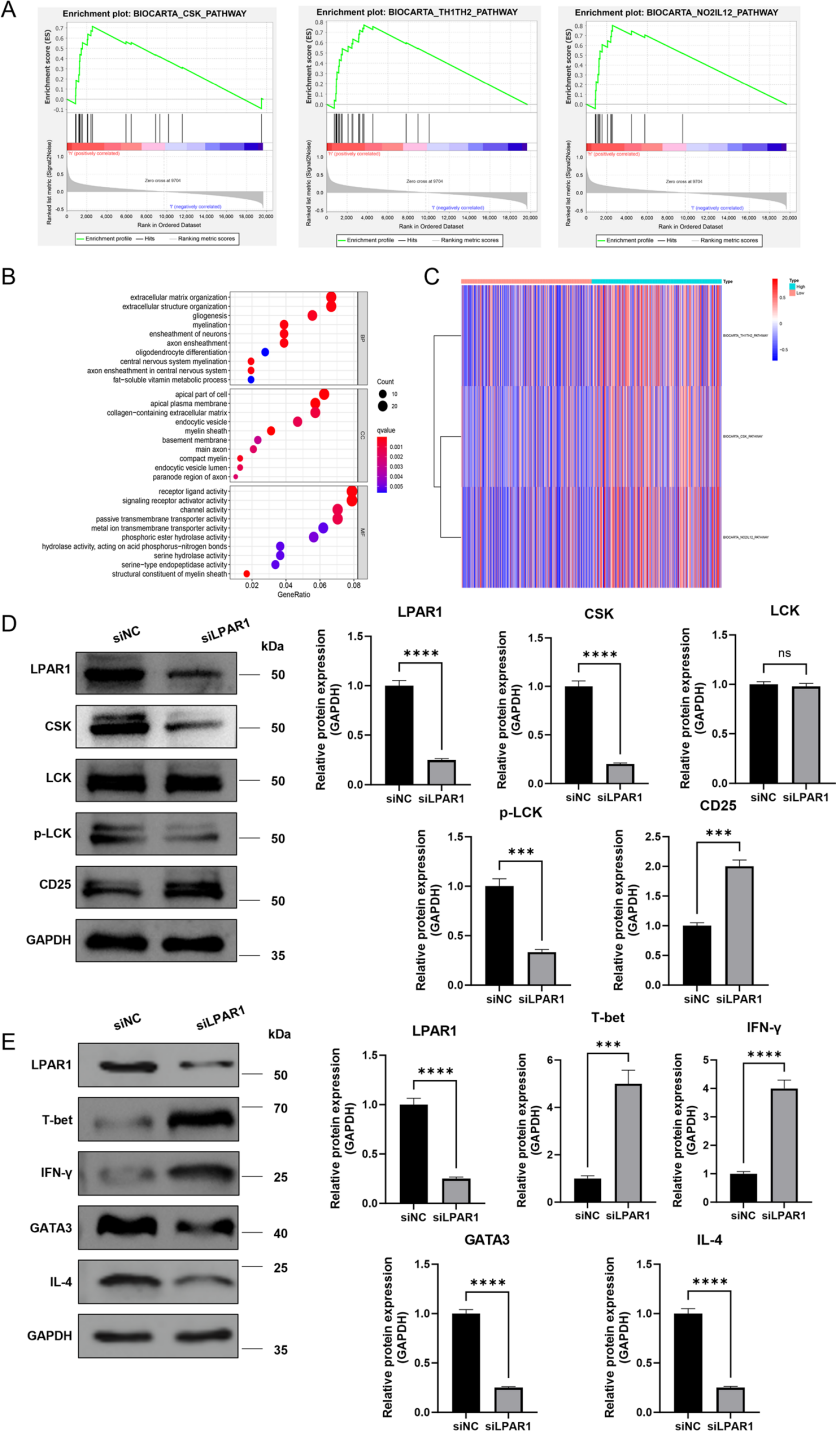
Bioinformatics analysis and western blot showed that LPAR1 regulated the immune signal pathways in LGG. **B** LPAR1-related biological processes in LGG by GO analysis. Enrichment plots from gene set enrichment analysis. **A** High-LPAR1 group was significantly enriched in immune related pathways; **C** Heat map of GSVA analysis. **D** CSK pathway genes were detected by Western blot in SW1088 cells. **E** Th1/Th2 pathway genes were detected by Western blot in SW1088 cells. Student’s *t*-test was employed for (**D** and **E**). Data are presented as mean ± SD, *P < 0.05, ** *P* < 0.01, *** *P* < 0.001, **** *P* < 0.0001, ns: no significance.

The enriched GO terms were classified into CC, BP and MF ontologies. To better understand the function role of LPAR1 in LGG, we conducted GO for the 417 DEGs (Fig. 5B). The results of BP analysis showed that DEGs were mainly enriched in development of nervous system, including oligodendrocyte differentiation, axon ensheathment, ensheathment of neurons, myelination. CC analysis revealed that the DEGs were significantly enriched in compact myelin, main axon, basement membrane, myelin sheath, endocytic vesicle. As for the MF, the DEGs were enriched in hydrolase activity, serine hydrolase activity, hydrolase activity, acting on acid phosphorus − nitrogen bonds, phosphoric ester hydrolase activity. Then, we performed GSEA between the low and high LPAR1 expression datasets to determine the differentially activated signaling pathways in LGG. We selected the most significantly enriched signaling pathways based on the NES. It showed that TH1TH2 pathway, CSK pathway, NO2/IL12 pathway were enrichment in LPAR1 high expression group (Fig. 5A). GSVA confirmed that these immune related pathways were indeed different between high and low LPAR1 expression groups (Fig. 5C). Accordingly, we examined the expression of CSK pathway genes by western blot, and the results showed that knockdown of LPAR1 decreased CSK, p-LCK, and upregulated CD25 expression (Fig. 5D). Similarly, T-bet and IFN-γ were upregulated while GATA3 and IL-4 were downregulated in the Th1/Th2 pathway (Fig. 5E).

### Low expression of LPAR1 inhibited the biological behavior of glioma cells

**Fig. 6:**
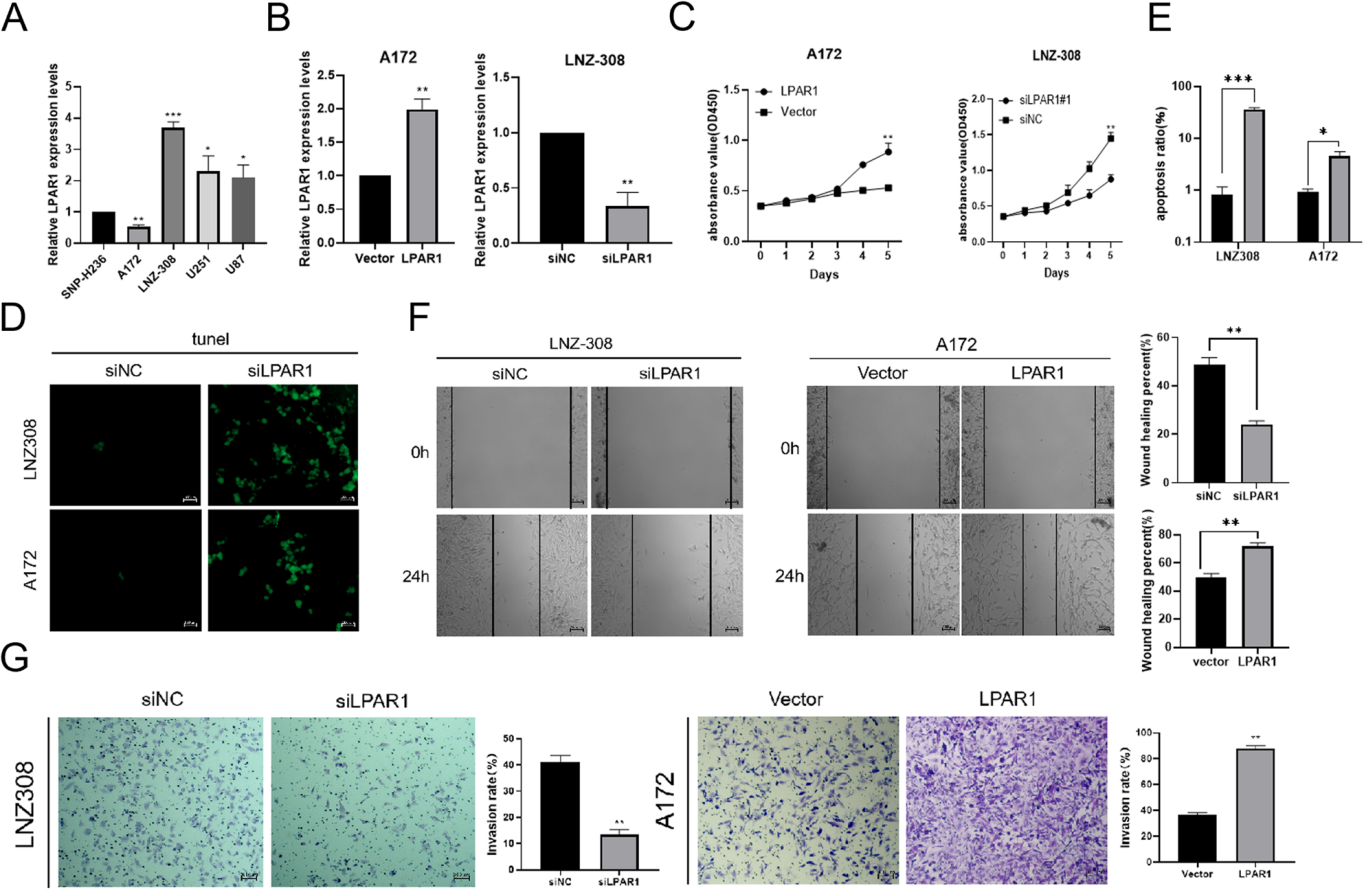
The impact of LPAR1 on the biological progression of GBM cell lines. **A** Quantitative Real-Time PCR (RT-qPCR) was employed to assess the differential expression of LPAR1 mRNA in human neuroglial cells (SNP-H236) and four representative glioblastoma multiforme (GBM) cell lines, namely A172, LNZ-308, U251, and U87. **B** The efficacy of LPAR1 overexpression and knockdown was confirmed via Quantitative Real-Time PCR (qRT-PCR). **C** Cell proliferation assays, using the CCK-8 method, were performed to evaluate the impact of LPAR1 on glioma cell proliferation. **D** TUNEL assays were conducted to assess the role of LPAR1 in regulating glioma cell apoptosis. **E** Statistical analysis of the apoptosis rate was performed on TUNEL-positive cells. **F** A wound-healing assay was employed to investigate the effect of LPAR1 on the migration capacity of glioma cells. **G** Transwell invasion assays were used to examine the influence of LPAR1 on the invasive potential of glioma cells. One-way ANOVA was employed for (**A**) and Student’s *t*-test was employed for (**B**, **C**, **E**, **F** and **G**). Data are presented as mean ± SD, *P < 0.05, ** *P* < 0.01, *** *P* < 0.001.

To investigate the biological role of LPAR1 in inhibiting glioma, we conducted a series of experiments to evaluate its function in various cellular processes. First, we used quantitative real-time PCR (RT-qPCR) to assess the differential expression of LPAR1 mRNA in human neuroglial cells (SNP-H236) and four representative glioblastoma multiforme (GBM) cell lines (A172, LNZ-308, U251, and U87, Fig. 6A). Next, we confirmed the overexpression and knockdown efficiency of LPAR1 using quantitative real-time PCR (qRT-PCR), ensuring successful modulation of LPAR1 expression in glioma cells for subsequent functional analysis (Fig. 6B). To evaluate the impact of LPAR1 on glioma cell proliferation, we performed CCK-8 cell proliferation assays (Fig. 6C). The results revealed that LPAR1 knockdown significantly inhibited glioma cell proliferation, while LPAR1 overexpression promoted cell proliferation, indicating that LPAR1 acts as an activator of glioma cell growth. We then conducted TUNEL assays to assess the role of LPAR1 in regulating glioma cell apoptosis. Knockdown of LPAR1 led to a significant increase in apoptosis in glioma cells (Fig. 6D). This suggests that knockdown of LPAR1 promotes apoptosis in glioma cells. Statistical analysis of TUNEL-positive cells confirmed that LPAR1 knockdown significantly enhanced the apoptosis rate, further supporting its anti-apoptotic role (Fig. 6E). To investigate the effect of LPAR1 on glioma cell migration and invasion, we performed wound-healing and Transwell invasion assays. The wound-healing assay demonstrated that LPAR1 knockdown significantly inhibited the migration of glioma cells, while LPAR1 overexpression promoted increased migration, suggesting that LPAR1 plays a key role in promoting cell migration (Fig. 6F). Furthermore, Transwell invasion assays revealed that LPAR1 knockdown significantly reduced the invasive potential of glioma cells, whereas LPAR1 overexpression led to increased invasion, highlighting LPAR1’s involvement in promoting glioma cell invasion (Fig. 6G). These findings underscore the potential of LPAR1 as a therapeutic target for the treatment of glioma.

**Fig. 7:**
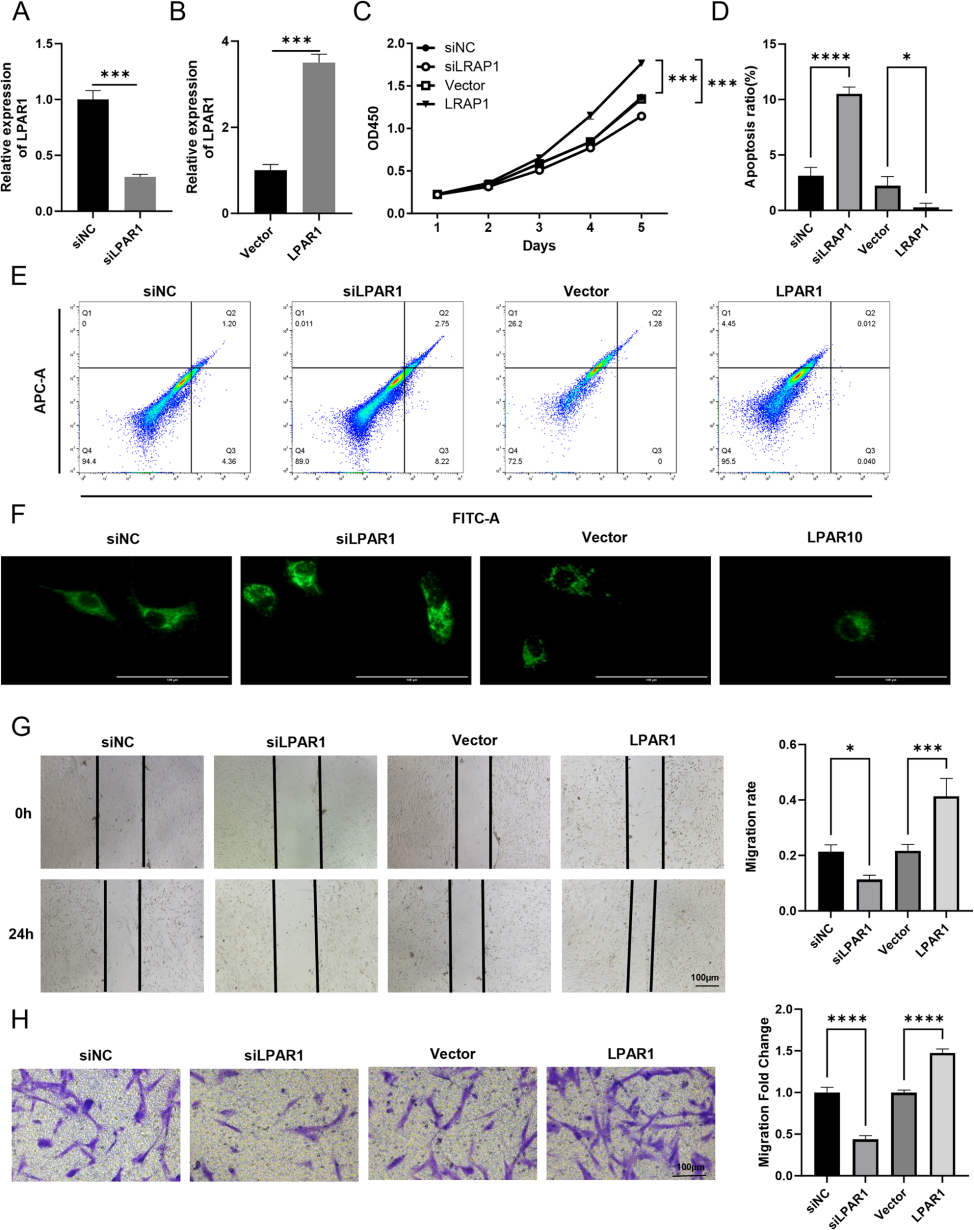
The impact of LPAR1 on the biological progression of LGG cell lines. Quantitative Real-Time PCR (RT-qPCR) was employed to assess the expression of LPAR1 mRNA in SW1088 cells after transfection with siLPAR1 (**A**) or LPAR1 plasmid (**B**). **C** Cell proliferation assays, using the CCK-8 method, were performed to evaluate the proliferation on SW1088 cells. Cell apoptosis detected by flow cytometry (**D**) and statistical graph. **F** TUNEL assays were conducted to assess the role of LPAR1 in cell apoptosis. **G** A wound-healing assay was employed to investigate the effect of LPAR1 on the migration capacity of SW1088 cells. **H** Transwell invasion assays were used to examine the influence of LPAR1 on the invasive potential of SW1088 cells. Student’s t-test was employed for (**A** and **B**) and One-way ANOVA was employed for (**C**, **D**, **G** and **H**). Data are presented as mean ± SD, *P < 0.05, ** *P* < 0.01, *** *P* < 0.001, **** *P* < 0.0001.

To confirm these results more reliably, we repeated the validation in LGG SW1088 cells. LPAR1 was knocked down or highly expressed, respectively, and confirmed by RT-qPCR (Fig. 7A, B). LPAR1 knockdown inhibited cell proliferation and promoted apoptosis (Fig. 7C-F). Accordingly, cell migration and invasion were also inhibited (Fig. 7G, H). However, upon high expression of LPAR1, these malignant behaviors were promoted and apoptosis was reduced. 

## Discussion

In this study, we found that the expression of LPAR1 was decreased in LGG tissues, and there was a negative correlation between LPAR1 and its methylation level. Furthermore, our study also found that different immune markers and TIICs levels were related to the distinctive expression of LPAR1 in LGG. Overexpression of LPAR1 in cell lines can promote the survival, proliferation and invasion of gliomas. Therefore, LPAR1 was a potential biomarker for predicting tumor prognosis and may have a potential impact on tumor immunity. Recent studies have shown that LGG shortens survival [29], which means that defining prognostic markers is an urgent need. Many genes have been identified as biomarkers for glioma in previous studies. For example, IDH1/2 wildtype is the primary WHO marker for high-grade glioma diagnosis, and its mutation with 1p/19q co-deletion is a molecular marker for oligodendrogliomas [21] MGMT is the most classical marker for chemosensitivity, which determines the choice of efficacy and treatment intensity [30]. In this study, we found that lower LPAR1 expression is associated with better patient survival in LGG patients. Stratified analysis showed that LPAR1 could accurately predict the survival outcome of different clinicopathological characteristics. Multivariate analysis further confirmed that LPAR1 was an independent prognostic factor for LGG. ROC curve (the AUC at 1 years, 3 years, 5 years were 0.687, 0.620 and 0.553) and meta-analysis (HR, 1.06; 95% CI, 0.95–1.17) indicated that LPAR1 was a powerful prognostic molecule for OS in LGG patients. LPAR1 significantly improved the predictive performance of a clinical model for LGG prognosis (Likelihood ratio test *P* = 0.002). IDH1 was considered to be an independent predictor of diffuse low-grade gliomas [31], but prognostic molecular markers for the non-mutated subgroup of IDH1 have not been reported. We found that LPAR1 was highly expressed in IDH wildtype, which highlights the potential of LPAR1 as a new biomarker. Indeed, previous studies have confirmed the different roles of LPAR1 in a variety of cancers. For example, in prostate cancer, LPAR1 was shown to be a potential prognostic biomarker, and high LPAR1 expression was associated with favorable overall survival [16]. In thyroid cancer, down-regulation of LPAR1 increases the invasive behavior of cancer cells [32]. Whereas in breast cancer, LPAR1 activates cancer stemness genes, leading to adriamycin resistance, and targeting the LPA-LPAR1 axis hinders the progression of metastatic breast cancer [33, 34] Silencing LPAR1 in ovarian serous cystadenocarcinoma inhibits tumor cell biological function and tumor formation in vivo [17]. LPAR1 appears to have a dual role in cancer. In this study we found that relatively high expression of LPAR1 in LGG was associated with worse prognosis, highlighting the unique value of LPAR1 in the prognostic diagnosis of LGG. Low expression of the gene may be a trigger for the initial onset, but at later stages of development, tumor cells may counteract stress or gain a survival advantage by increasing its expression. Consistent with our findings, in glioblastoma, LPA1 causes PKCα nuclear translocation and promotes tumor growth [35].

The effects of epigenetic modifications on gene expression have been increasingly scrutinized. For example, m⁶A modification of SNAP29 mRNA decreases its protein level [36]. Aberrant methylation exists in a multilevel regulatory pattern in glioma development [37, 38]. Evidence suggested that m^6^A-mediated upregulation of the lncRNA CHASERR, an epitranscriptional regulation, was shown to promote glioma progression [39]. In this study, we analyzed the correlation between the methylation of LPAR1 and its expression, and except for cg09549894, all other methylation sites were significantly negatively correlated with LPAR1 expression. Most of these sites were associated with overall survival (OS) or progression-free survival (PFS) in LGG patients, and low methylation levels predicted a worse prognosis. By in vitro methylation and dual-luciferase reporter assay, we confirmed that methylation of LPAR1 inhibits its expression. These results suggest that the methylation level of LPAR1 predicts the survival of LGG patients, which further highlights the prognostic value of LPAR1 in LGG. Consistent with our findings, in gastric cancer, spearman correlation analysis showed that LPAR1 expression was negatively correlated with methylation levels, and cg14231369 and cg14429427 may be potential regulatory sites [40].

Imbalance of TIICs has become the key to tumor growth and progression in LGG [41, 42]. Previous studies have pointed out that the lncRNA was associated with tumor immune infiltration and immune checkpoints in LGG, and that high expression predicted a poorer prognosis [43]. In this study, we found that the expression of LPAR1 was positively correlated with the expression of B cells, CD8 + T cells, CD4 + T cells, macrophages, etc., and correlated with the marker genes of these immune cells. LPAR1 has been confirmed to be involved in the activation, proliferation, differentiation, and migration of immune cells [44], and could activate macrophages through LPA [45]. More importantly, high immune infiltration and high LPAR1 expression predicted the worst prognosis. In addition, we found that the number of CD4 + T and B cells decreased after the knockdown of LPAR1, and the content of CD19, IL-4 was reduced, which suggests that LPAR1 may have a potential regulatory effect on the LGG in the B and T cells, which was also verified by detecting the effect of LPAR1 on CSK pathway and Th1/Th2 pathway. In fact, consistent with our findings, several other studies have similarly demonstrated that increased TIICs, especially myeloid cells (neutrophils, macrophages, etc.) and T and B cells, is indeed associated with a worse prognosis in LGG [46–48]. This correlation was also found in other studies, where LPAR1 was positively correlated with the degree of infiltration of immune-infiltrating cells (including CD4 + T cells, B cells, etc.) in prostate and thyroid cancers [16, 33]. Notably, significant immune cell infiltration (T cells and macrophages) was also present in double negative astrocytopathy, accompanied by astrocytic loss [49]. These observations underscore that the mere presence of immune cells or their signature genes is insufficient to confer tumor specificity. Consequently, TIIC signatures in glioma should be interpreted as associative rather than pathognomonic markers of malignancy. Another important finding of this study was that LPAR1 knockdown inhibited the malignant behavior of a variety of glioma cell lines, as evidenced by diminished cell proliferation, migration, and invasion, along with increased apoptosis. These evidences initially indicate the anti-tumor effect of LPAR1 inhibition and provide a basis for further investigation of the role and mechanism of LPAR1 in LGG.

Nonetheless, this study has some limitations. First, the patient data used in this study were obtained from public databases and were retrospective, with no clinical data collected for further validation and no patient-derived tumor samples were obtained for immunohistochemistry testing of LPAR1. Second, in subgroup analyses, certain groups suffered from low sample sizes (e.g., IDH1 wildtype), although statistical differences have been demonstrated. In addition, although we experimentally validated key conclusions, such as the inhibition of LPAR1 expression by methylation and the effect of LPAR1 on TIICs (e.g., CD4 + T cells and B cells), and confirmed the effect of LPAR1 on tumor cell proliferation, migration, and other behaviors, these conclusions still need to be confirmed by more comprehensive prospective studies; besides, although we confirmed the relationship between low expression of LPAR1 in relation to survival and TIICS and preliminarily confirmed the regulatory effects of LPAR1 on immune cells and pathways, we have not yet been able to confirm a direct causation between LPAR1-regulated TIICs and prognosis due to the limitations of in vitro experiments. More importantly, the effect of LPAR1 inhibition on tumor growth and systemic safety still need to be confirmed in xenograft models. On the other hand, the relatively high and low methylation expression of LPAR1 in LGG predicts a worse prognosis for patients, suggesting its potential for risk stratification. Therefore, in the future, LPAR1 could be further stratified to investigate the predictive value of its expression for tumor staging and prognosis. Alternatively, precise multi-target combination therapy may provide better efficacy, for which LPAR1 could be integrated into existing staging systems, drawing on coupled models to evaluate synergistic effects and exploring its potential value when combined with existing markers such as IDH1/ and 1p19q, which would require additional prospective studies [50]. This approach holds promise for guiding treatment intensity, reducing toxicity, and maintaining efficacy.

In conclusion, the present study firstly identified low LPAR1 expression or high LPAR1 methylation expression as potential molecular markers of good prognosis in patients with low-grade glioma (LGG). The mechanism by which LPAR1 inhibits cancer progression may be related to the regulation of the proportion of TIICs in the tumor microenvironment (TME), a pattern that may act through the CSK, Th1/Th2 pathway. In glioma cell lines, knockdown of LPAR1 inhibited tumor biological behavior, and these findings provide new ideas for therapeutic and prognostic guidance in LGG.

## Supplementary Information

Supplementary Material 1.

Supplementary Material 2.

Supplementary Material 3.

## Data Availability

The data that support the findings of this study are available from the corresponding author, [LL], upon reasonable request.
