# Supplementary material for: Prognostic value of LPAR1 expression and methylation in low-grade gliomas: a meta-analysis of TCGA and CGGA datasets and functional validation
Source: BMC Cancer. 2025 Dec 30;26:161. doi: 10.1186/s12885-025-15406-z (PMC12859871; doi:10.1186/s12885-025-15406-z)
Supplement: Supplementary file 2 — Supplementary Material 2. [file 12885_2025_15406_MOESM2_ESM.docx]

**Supplementary information for manuscript “Prognostic value of LPAR1 expression and methylation in low-grade gliomas: a meta-analysis of TCGA and CGGA datasets and functional validation”**

**Supplementary Figures**


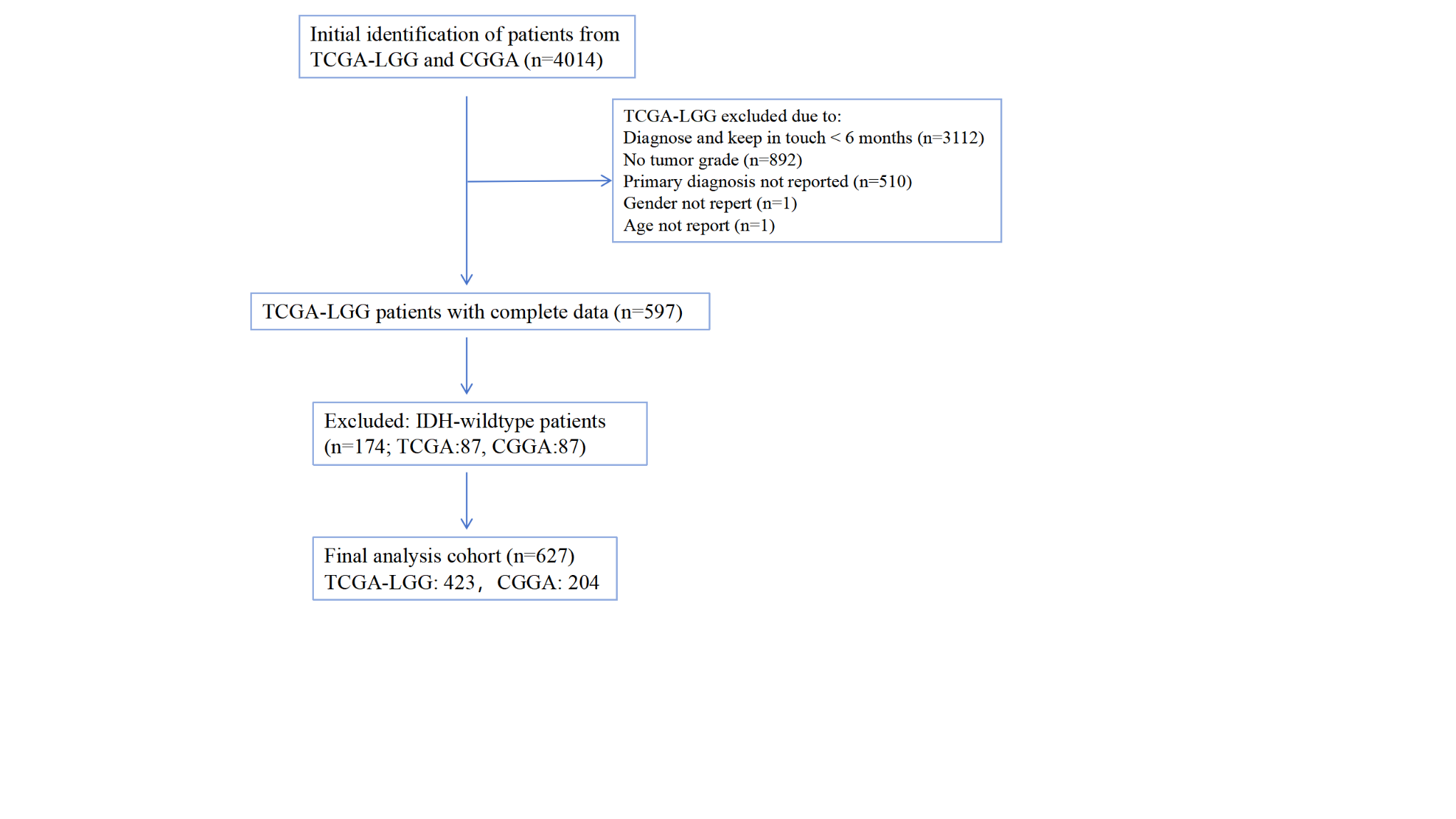


**Figure S1: Case screening process for the TCGA and CGGA database.**


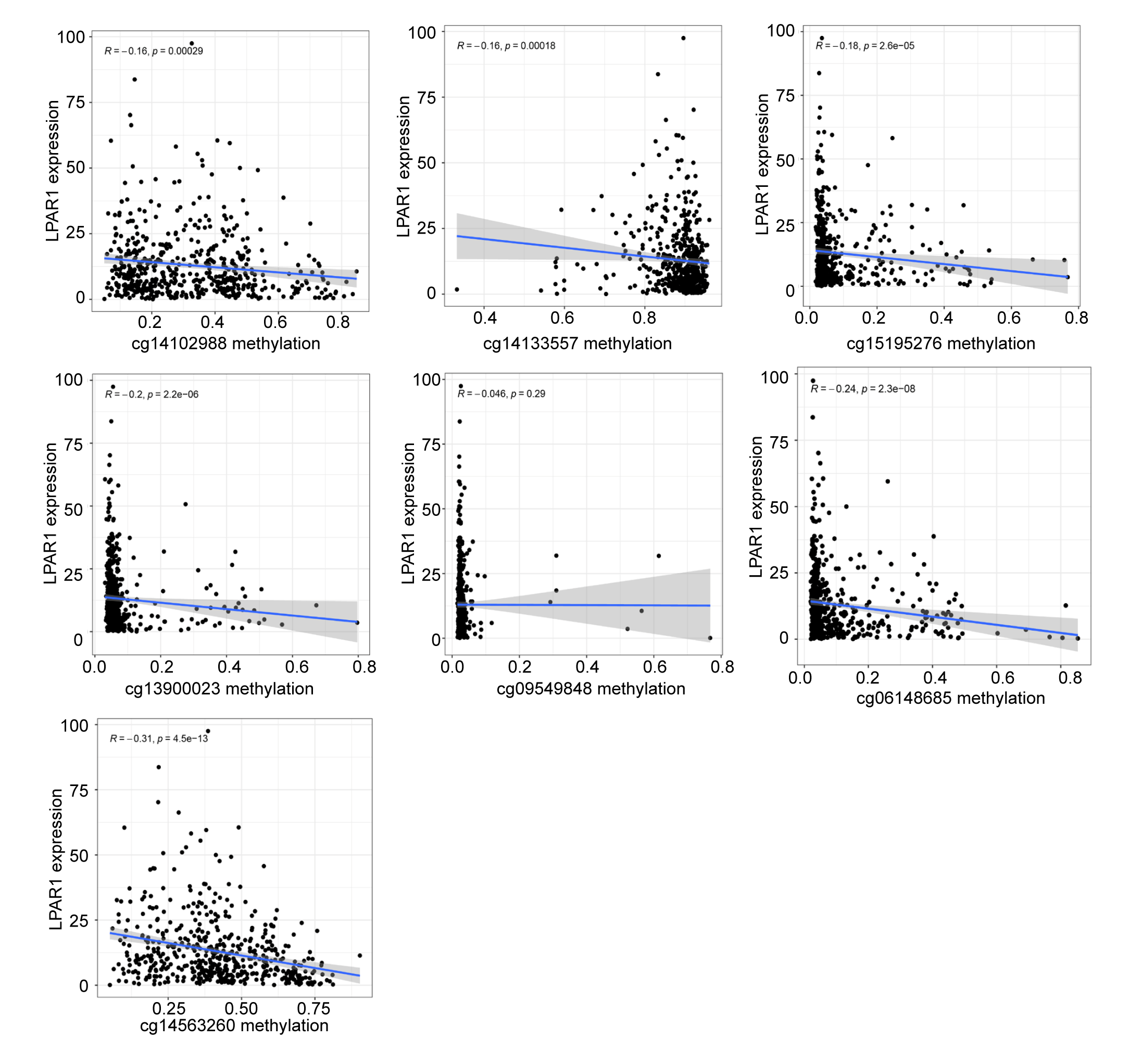


**Figure S2:** **Scatter plot of correlation between LPAR1 gene expression and methylation levels at different CpG sites.** The figure shows the results of correlation analysis between LPAR1 gene expression levels and methylation levels at several different CpG sites (cg14102988, cg14133557, cg15195276, cg13900023, cg09549848, cg06148685, cg14563260).


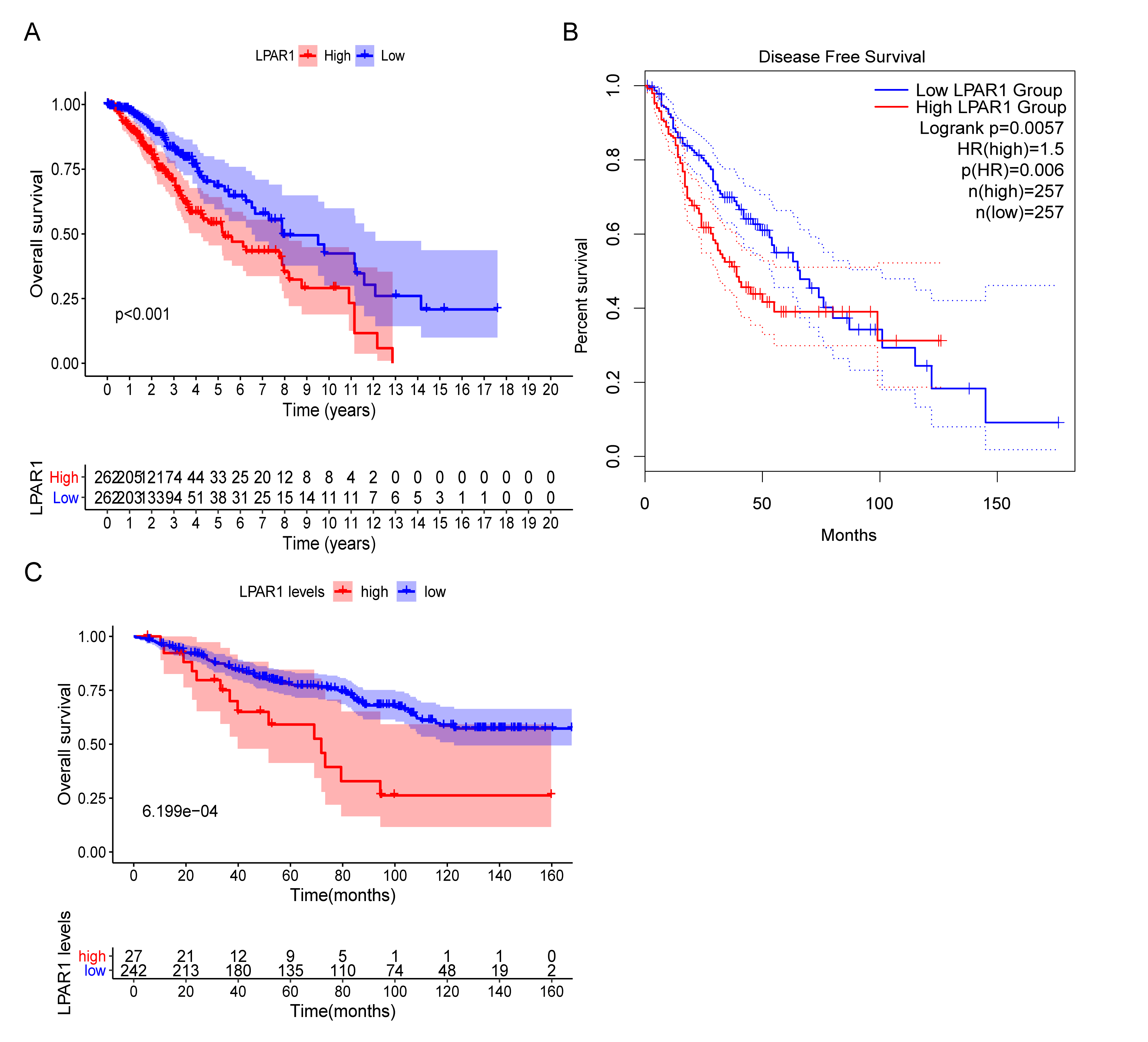


**Figure S3:** **Relationship between LPAR1 expression levels and patient survival prognosis.** Kaplan-Meier survival analysis of LPAR1 expression levels on patient (A) OS and (B) PFS on TCGA-LGG database. (C) Kaplan-Meier survival analysis of LPAR1 expression levels on patient OS on CGGA dataset. The blue curve is the LPAR1 low expression group, and the red curve is the LPAR1 high expression group.


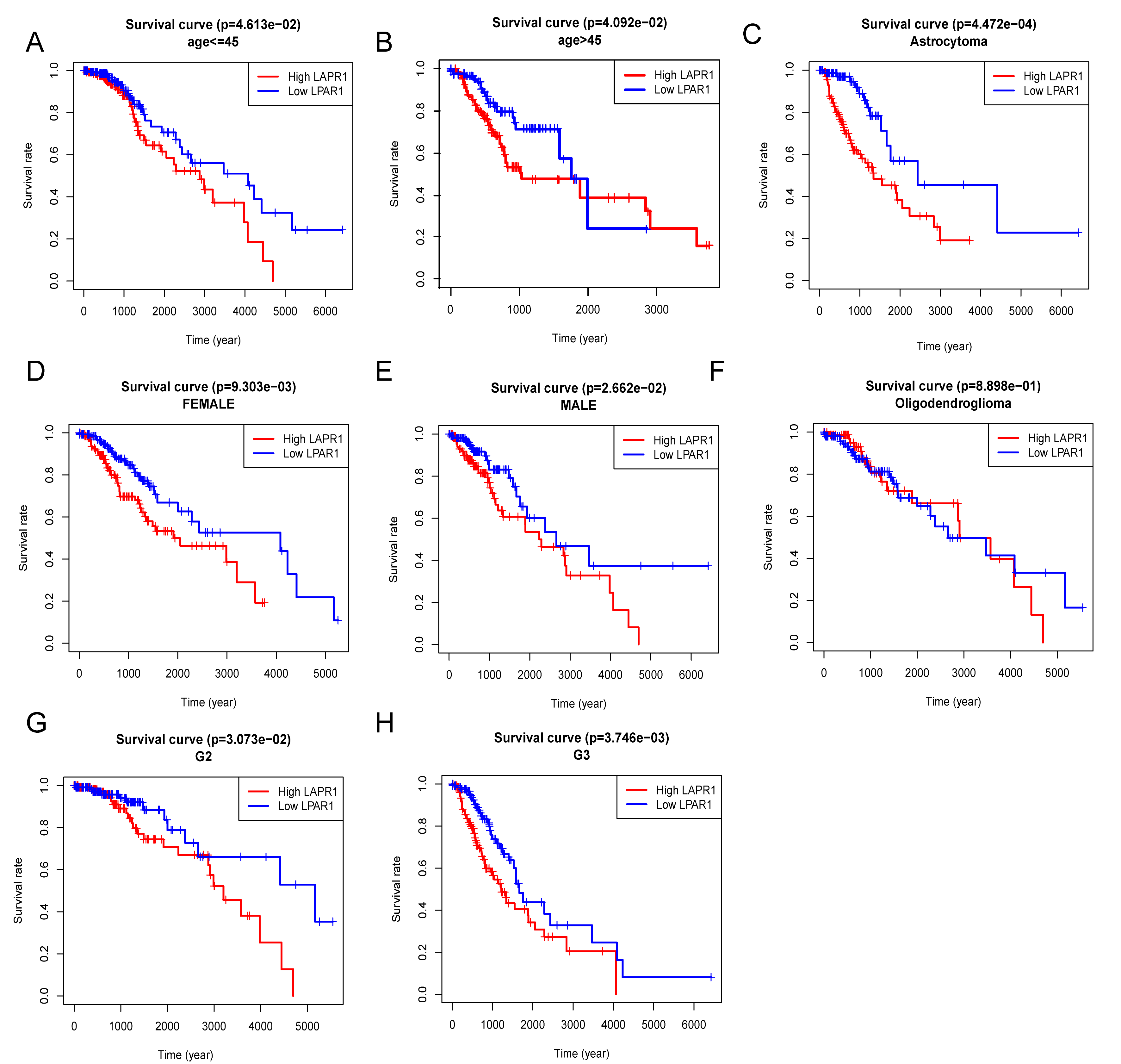


**Figure S4:** **Relationship between LPAR1 expression levels and patients' survival and prognosis stratified by different clinicopathologic features.** Kaplan-Meier survival curves for patients with (A) age ≤ 45, (B) age > 45, (C) astrocytoma, (D) female, (E) male, (F) oligodendroglioma, (G) G2 classification and (H) G3 classification in the group with high expression of LPAR1 (red curve) versus the group with low expression (blue curve).


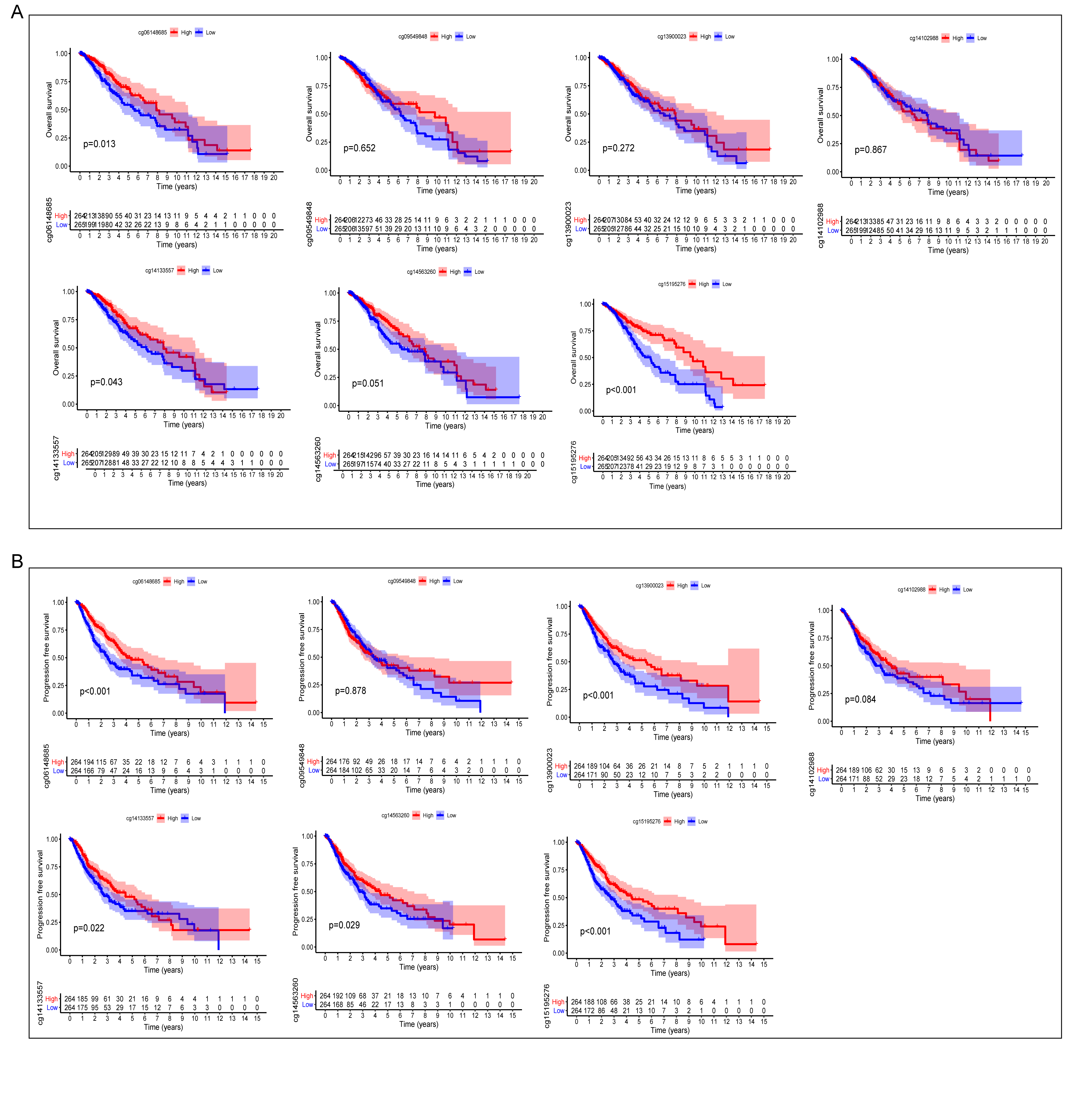


**Figure S5: Kaplan-Meier survival curves of different LPAR1 methylation regions including** (A) Relationship between methylation levels of 7 sites and OS in LGG patients. (B) Relationship between methylation levels of 7 sites and PFS in LGG patients.


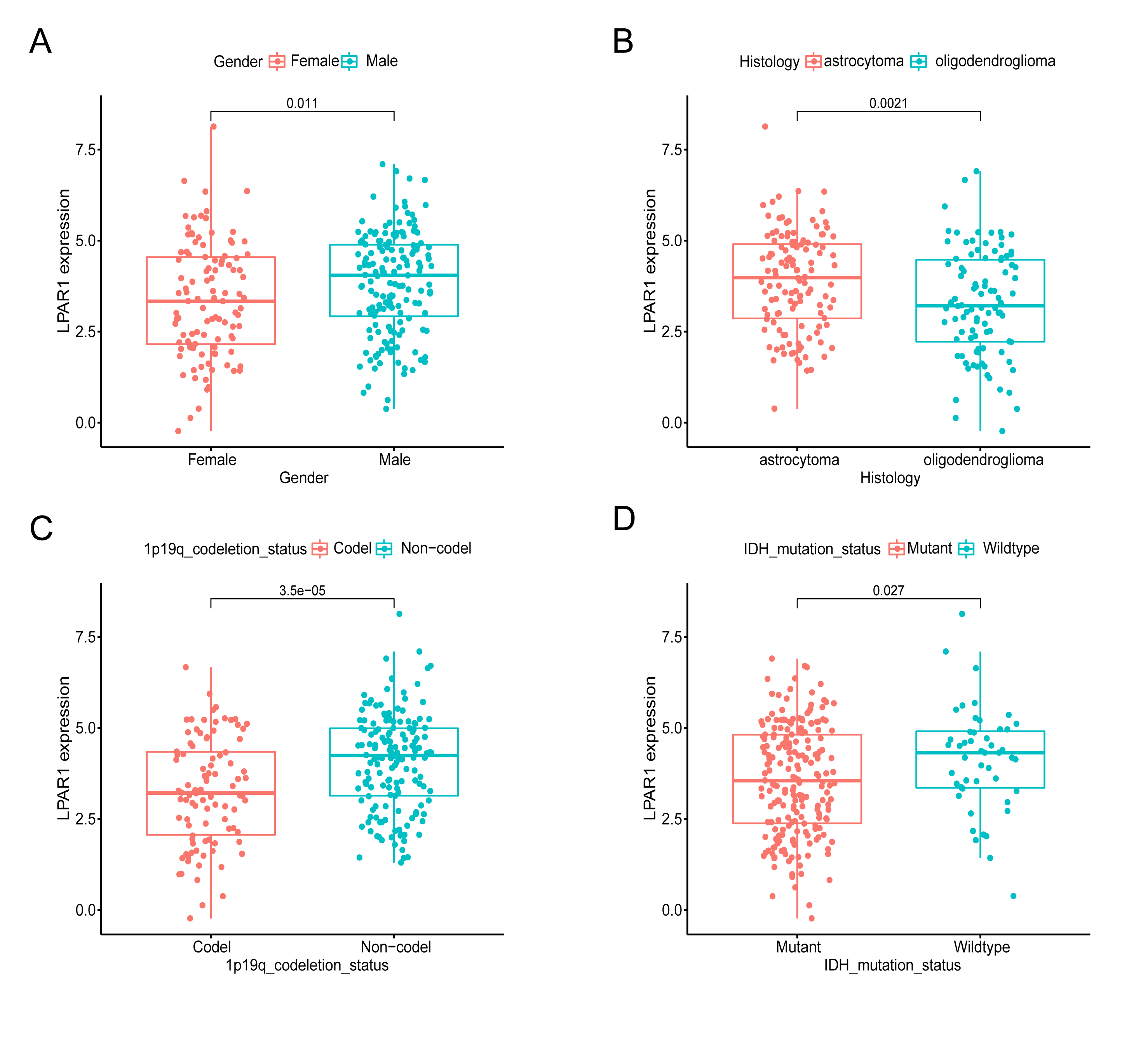


**Figure S6:** **Correlation analysis of LPAR1 gene expression levels under different clinicopathologic features in CGGA dataset.** The analysis compared the expression of LPAR1 in CGGA dataset according to (A) histological types; (B) IDH mutation status, (C) gender and (D) 1p19q codeletion status.


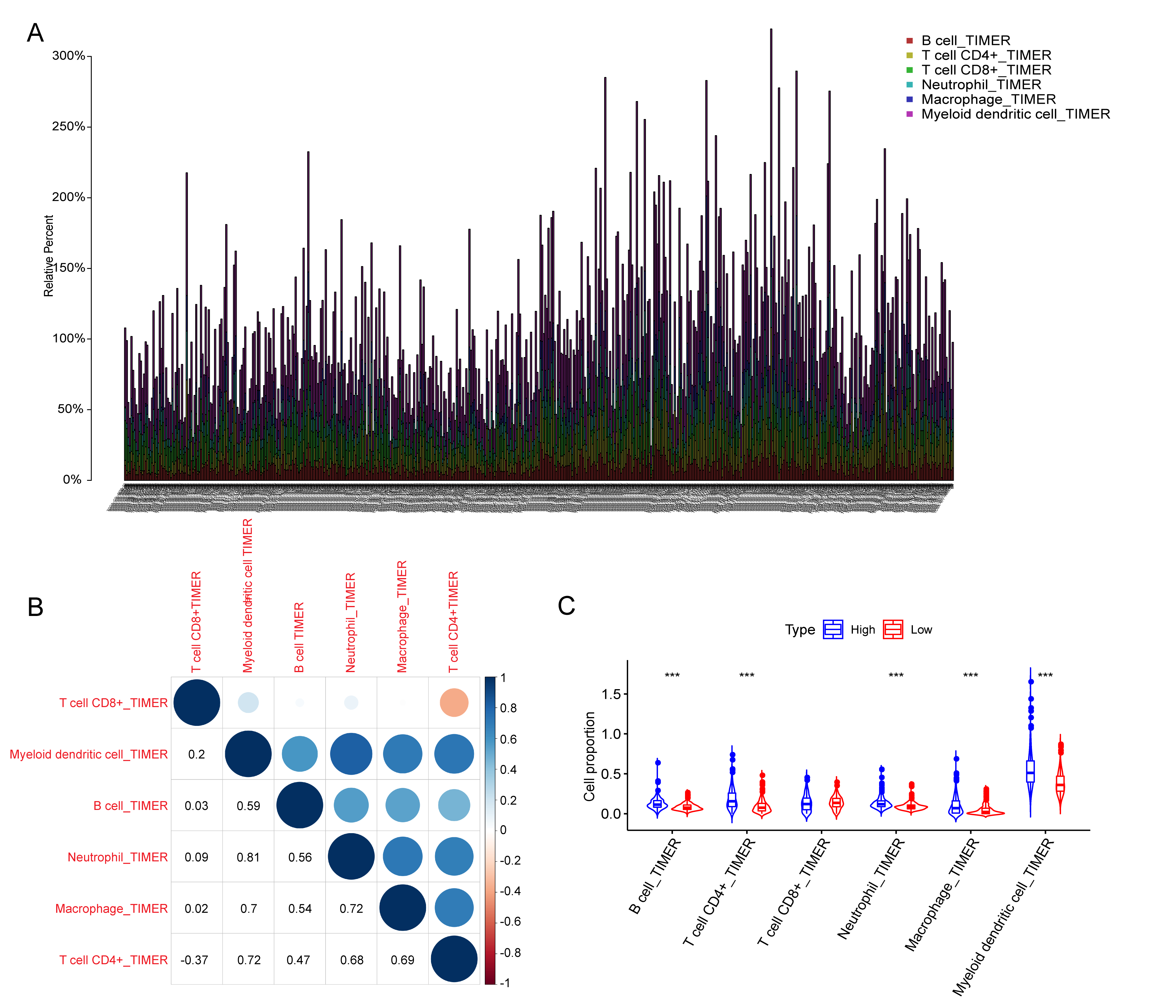


**Figure S7:** Low LPAR1 expression reduced the proportion of TIICs in the TME. (A) Proportion of 6 immune cells in LGG patients. (B) Correlation between immune cells. (C) Proportion of immune cells between high and low LPAR1 expression groups.


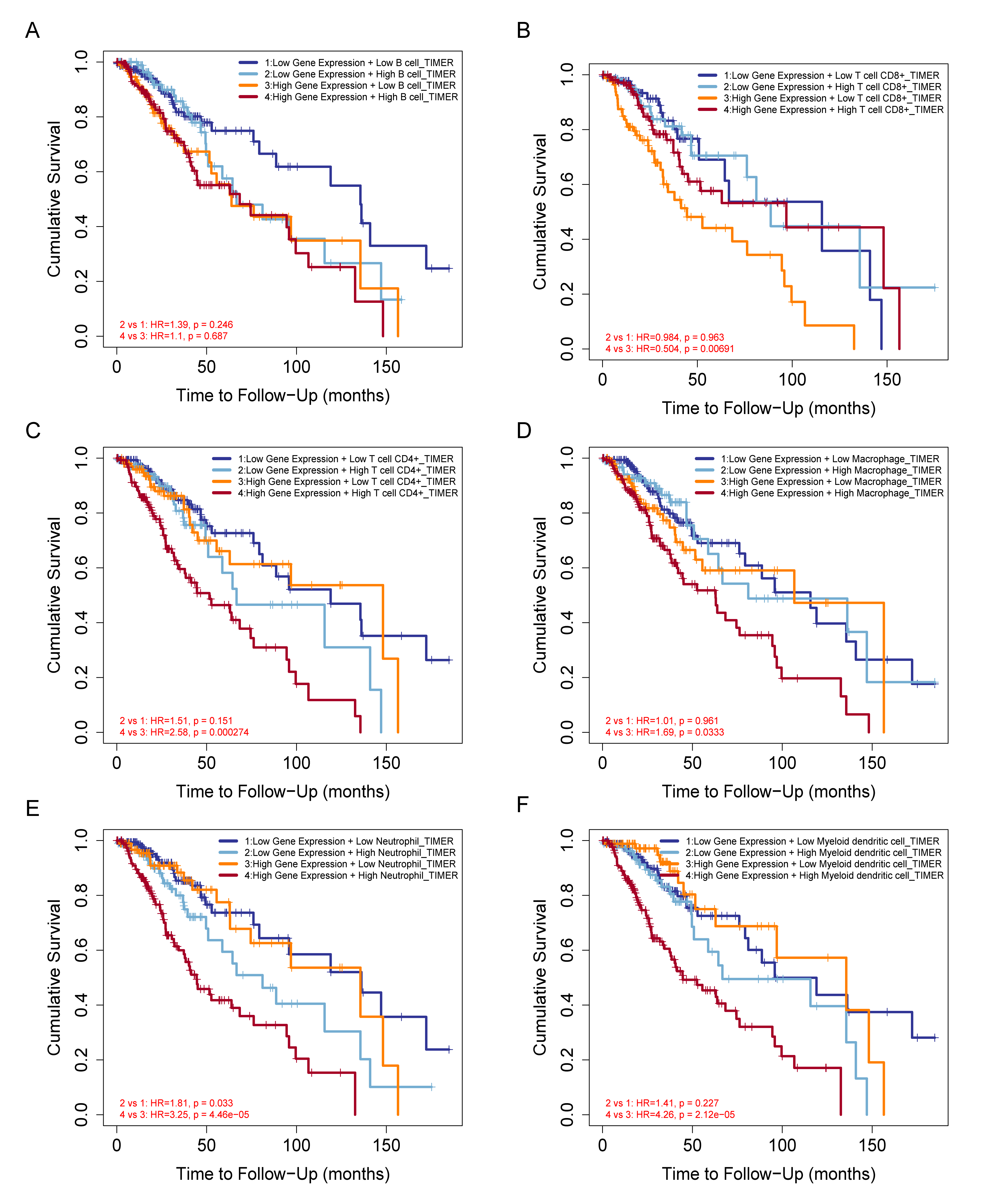


**Figure S8:** **Survival analysis based on LPAR1 gene expression levels grouped with immune cell infiltration levels.** Kaplan - Meier survival curves of patients grouped by LPAR1 gene expression levels in combination with levels of (A) B cells, (B) CD8+ T cells, (C) CD4+ T cells, (D) macrophages, (E) neutrophils, and (F) myeloid dendritic cell infiltration.
