# Supplementary material for: Prognostic value of LPAR1 expression and methylation in low-grade gliomas: a meta-analysis of TCGA and CGGA datasets and functional validation
Source: BMC Cancer. 2025 Dec 30;26:161. doi: 10.1186/s12885-025-15406-z (PMC12859871; doi:10.1186/s12885-025-15406-z)
Supplement: Supplementary file 3 — Supplementary Material 3. [file 12885_2025_15406_MOESM3_ESM.docx]

**Original blots of Western Blot**

**Figure 5D**

| 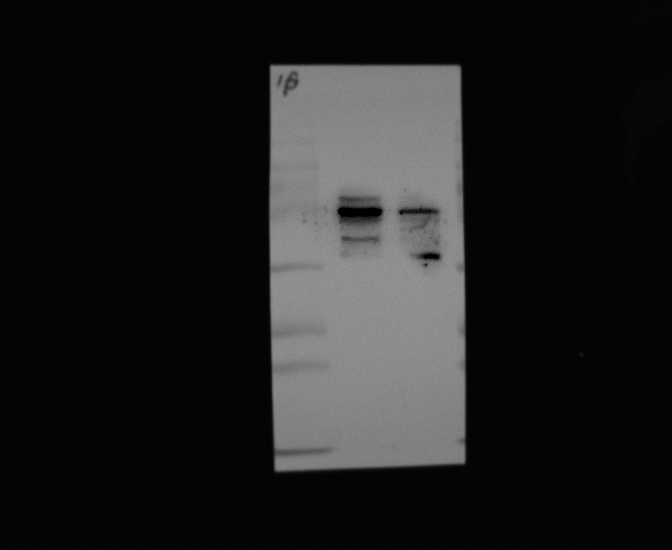 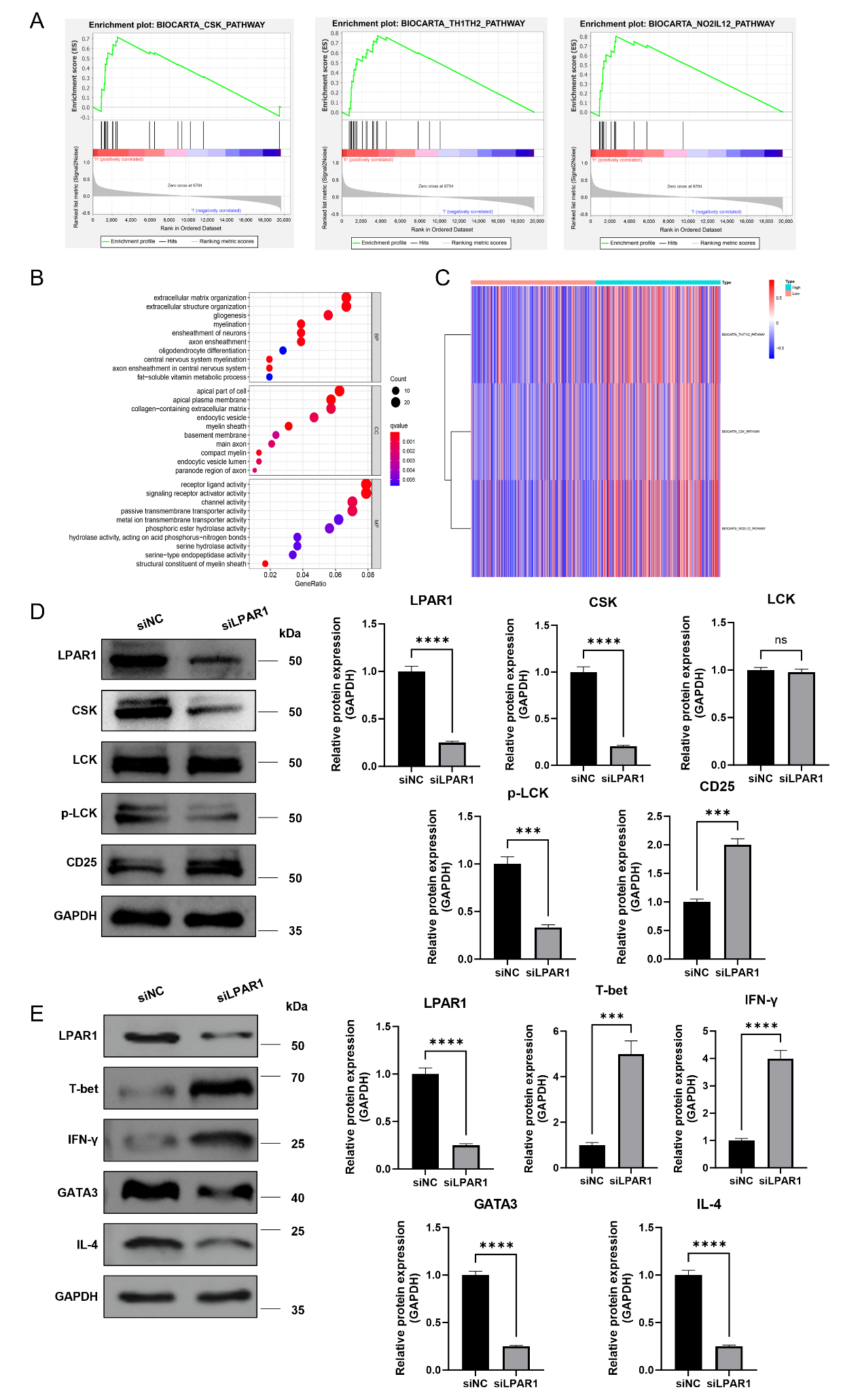  **LPAR1 50 kDa**  **siLPAR1**  **siNC** |
| --- |
| Extended Data Figure 1. The original blotting of LPAR1 in Figure 5D. Left, original blotting of LPAR1 and corresponding sample names; Right, cropped version in manuscript. |
| 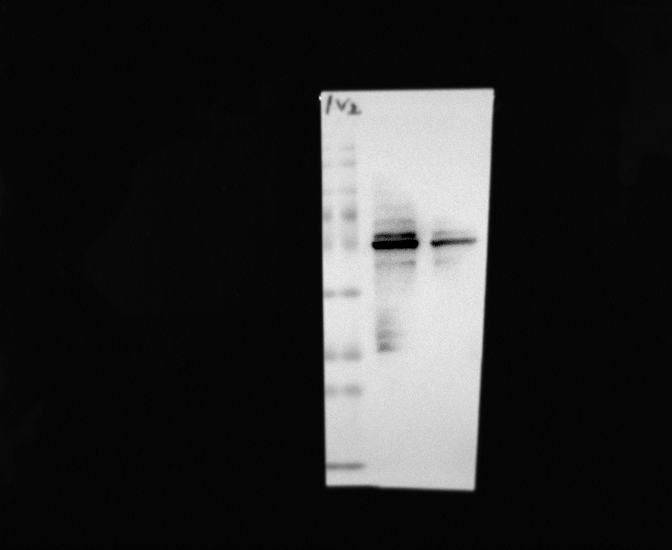 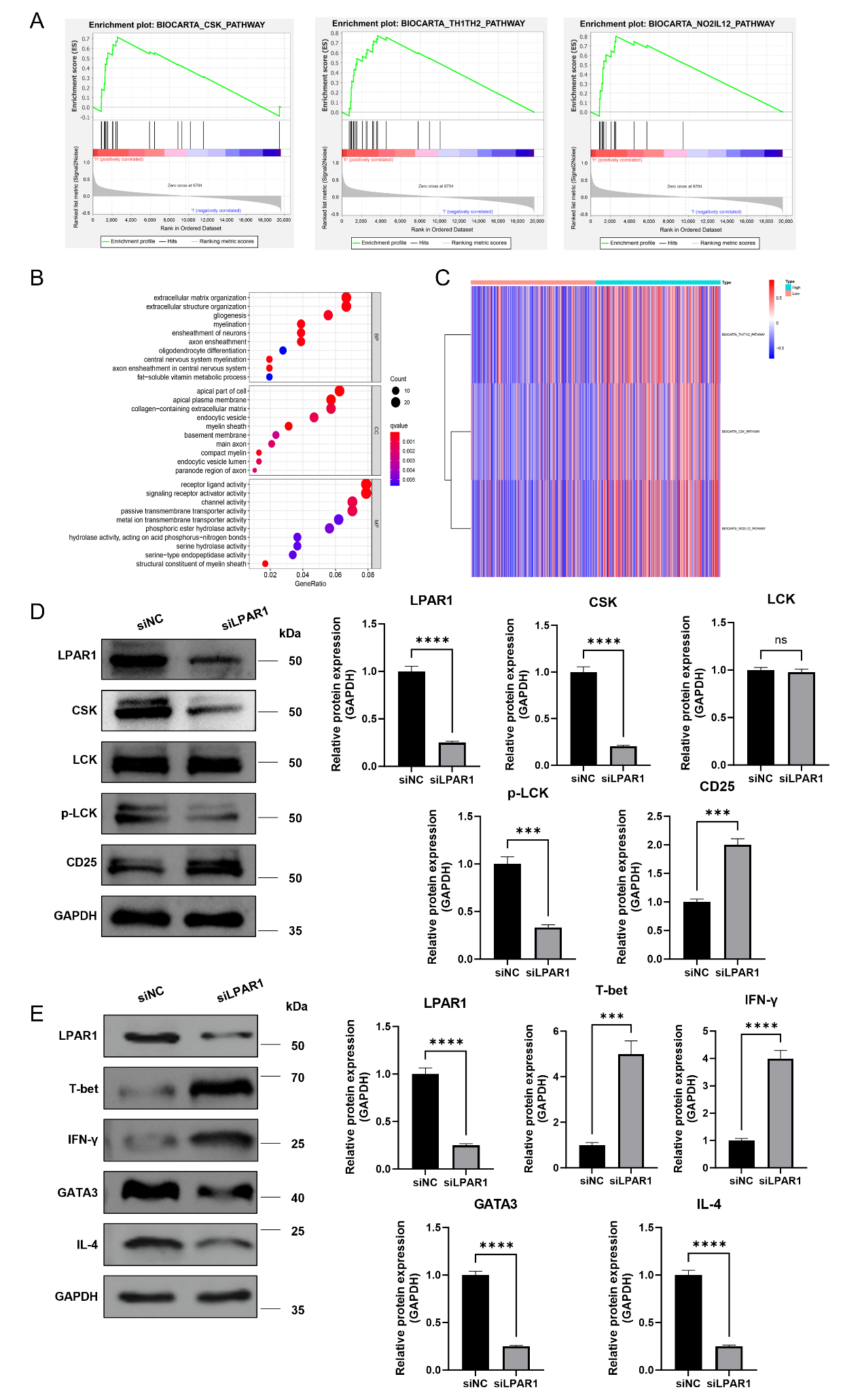  **CSK 50 kDa**  **siLPAR1**  **siNC** |
| Extended Data Figure 2. The original blotting of CSK in Figure 5D. Left, original blotting of CSK and corresponding sample names; Right, cropped version in manuscript. |
| 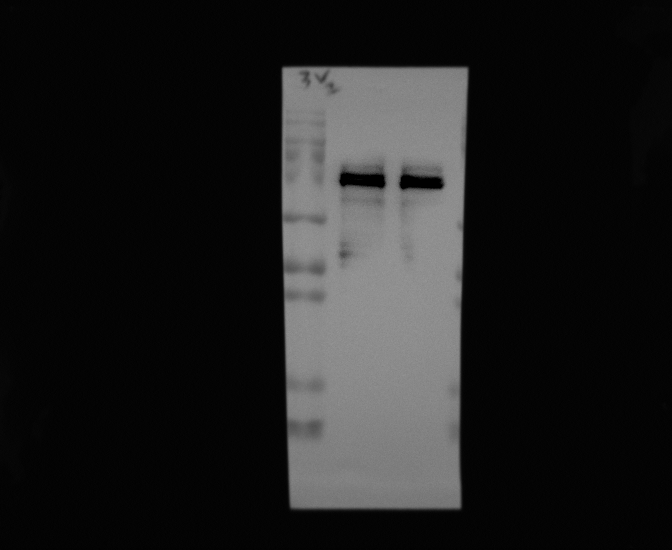 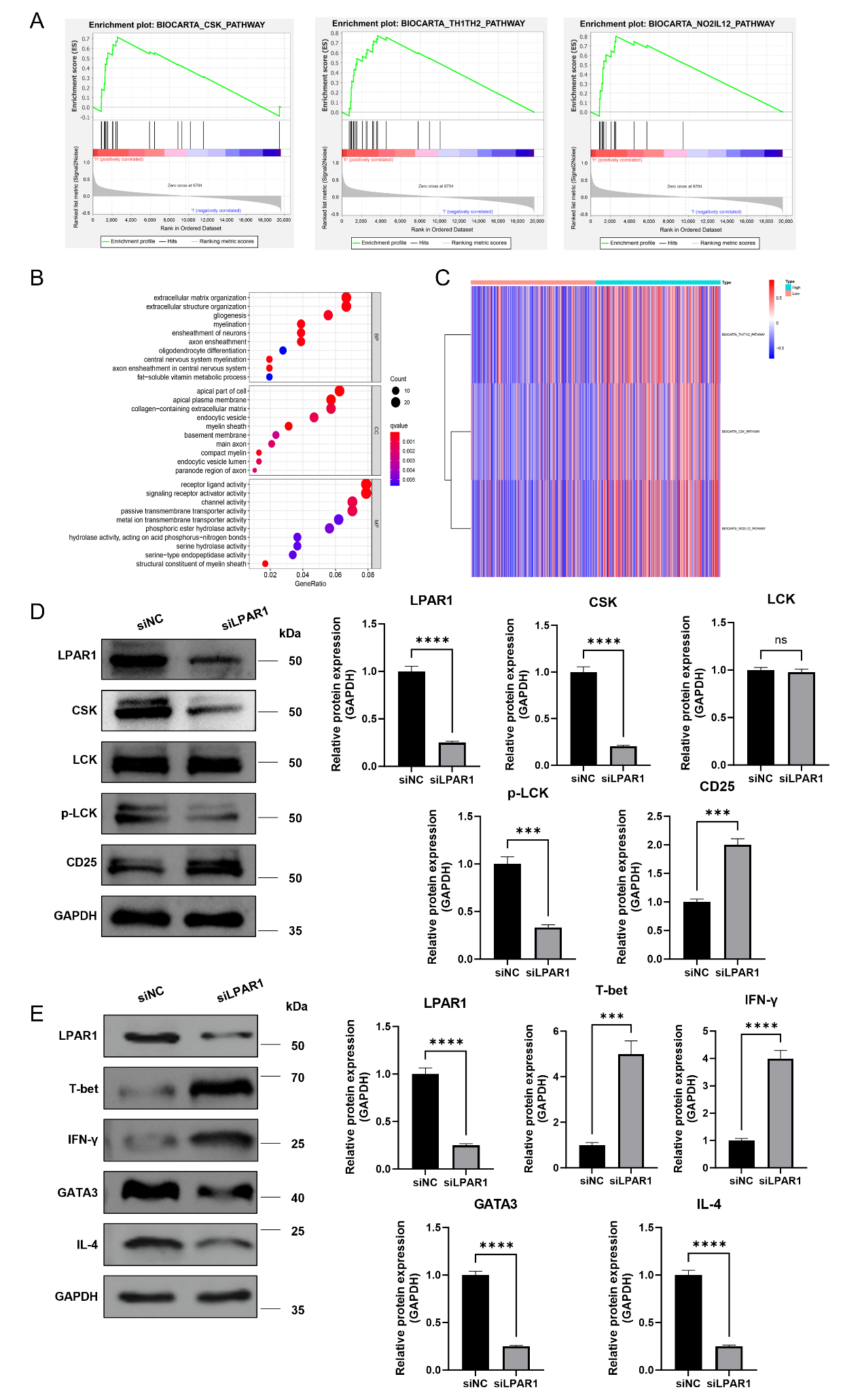  **LCK 50 kDa**  **siNC**  **siLPAR1** |
| Extended Data Figure 3. The original blotting of LCK in Figure 5D. Left, original blotting of LCK and corresponding sample names; Right, cropped version in manuscript. |
| 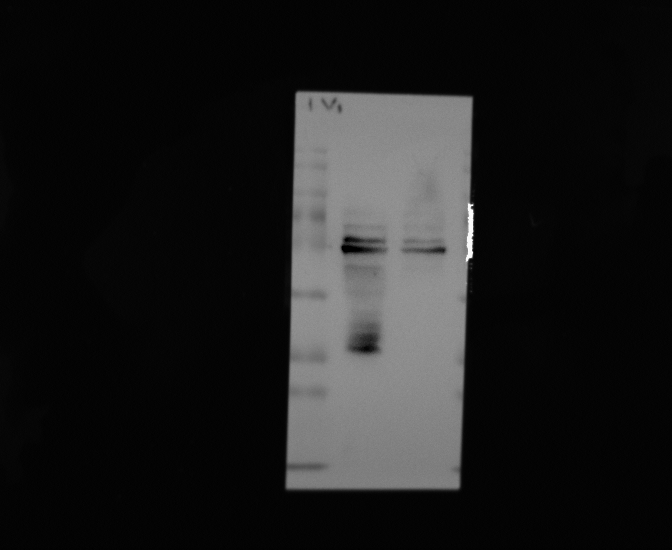 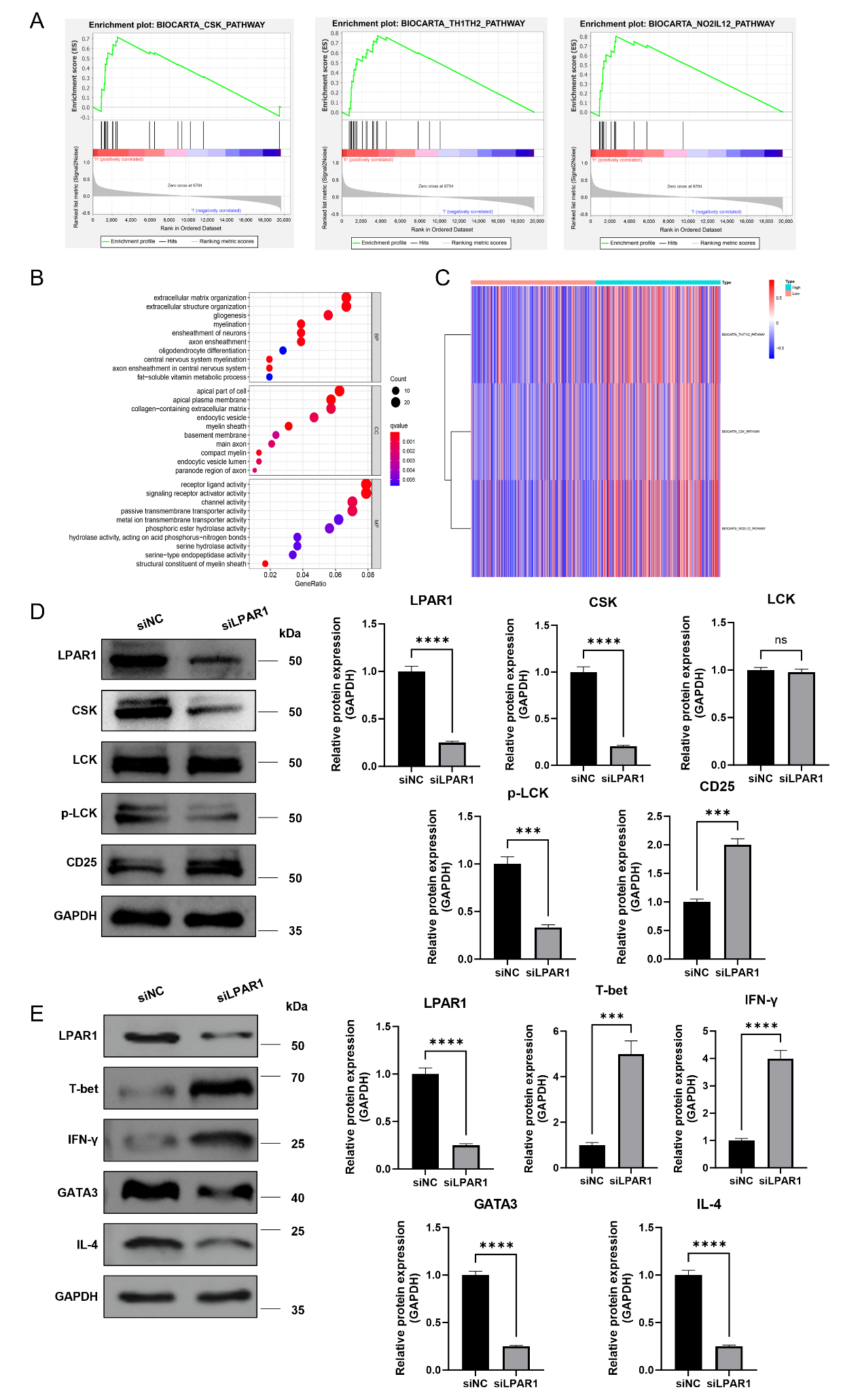  **p-LCK 50 kDa**  **siLPAR1**  **siNC** |
| Extended Data Figure 4. The original blotting of p-LCK in Figure 5D. Left, original blotting of p-LCK and corresponding sample names; Right, cropped version in manuscript. |
| 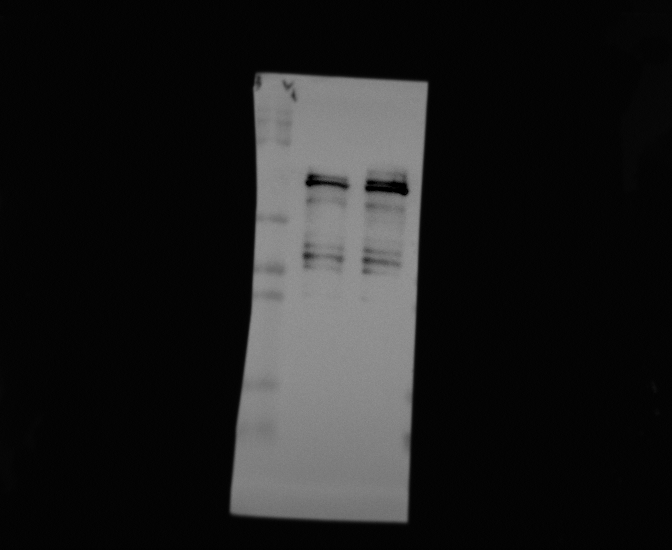 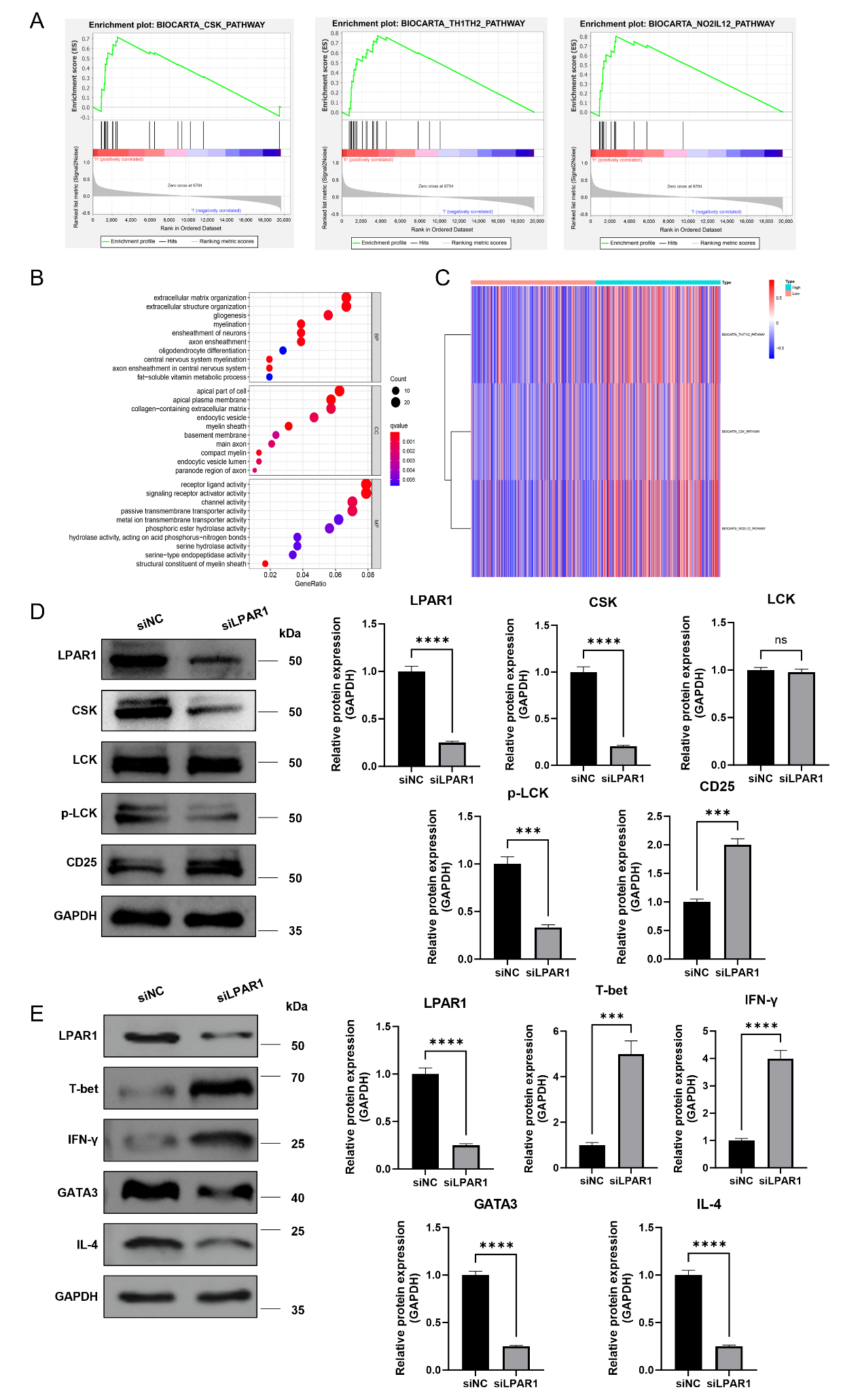  **CD25 65 kDa**  **siLPAR1**  **siNC** |
| Extended Data Figure 5. The original blotting of CD25 in Figure 5D. Left, original blotting of CD25 and corresponding sample names; Right, cropped version in manuscript. |
| 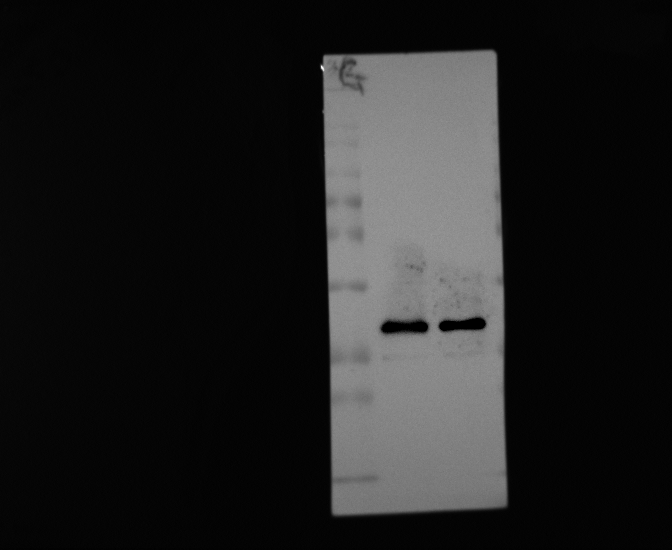 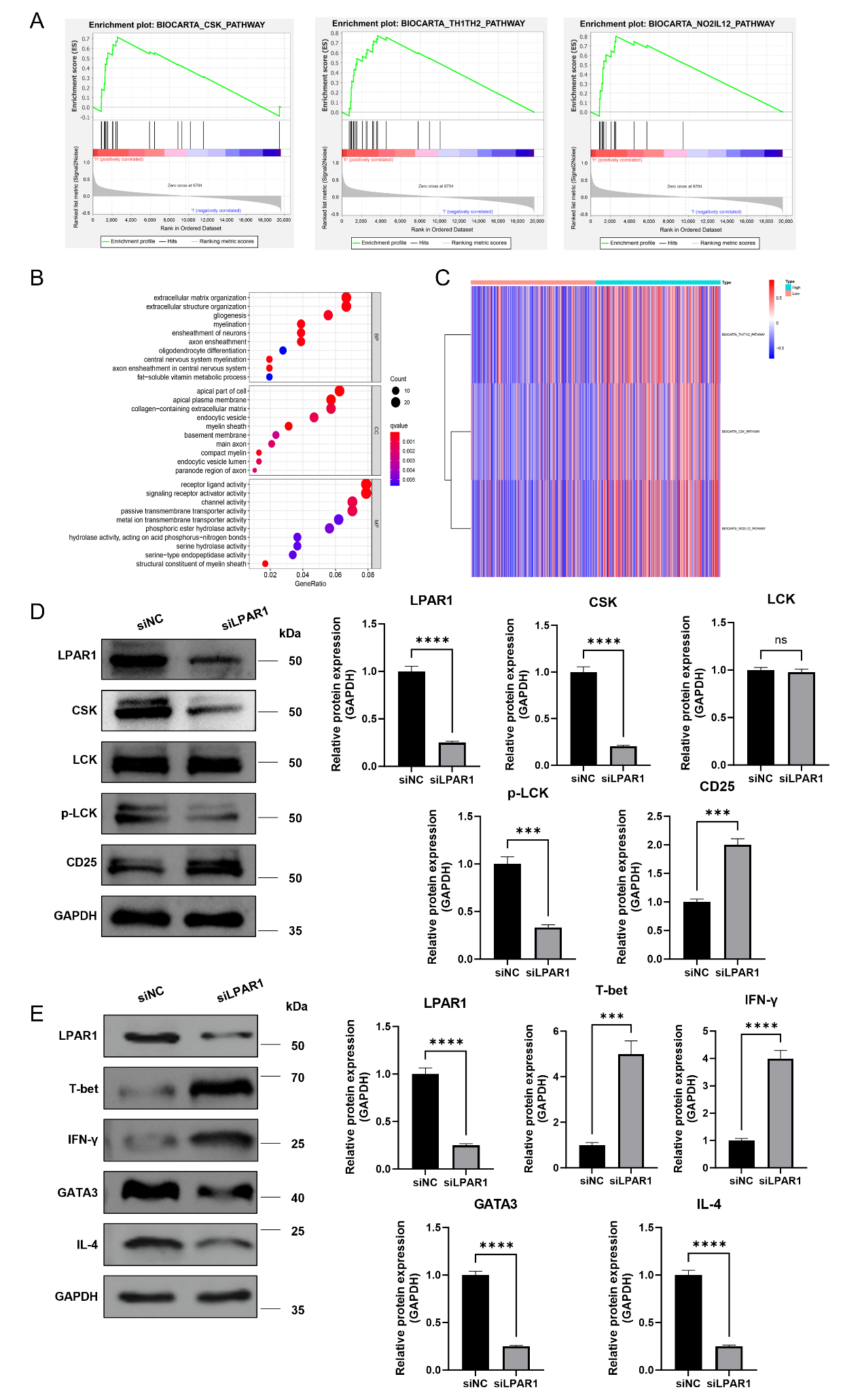  **siLPAR1**  **siNC**  **GAPDH 36 kDa** |
| Extended Data Figure 6. The original blotting of GAPDH in Figure 5D. Left, original blotting of GAPDH and corresponding sample names; Right, cropped version in manuscript. |

**Figure 5E**

| 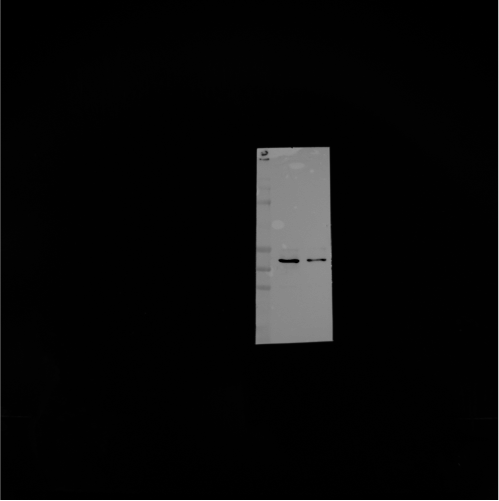 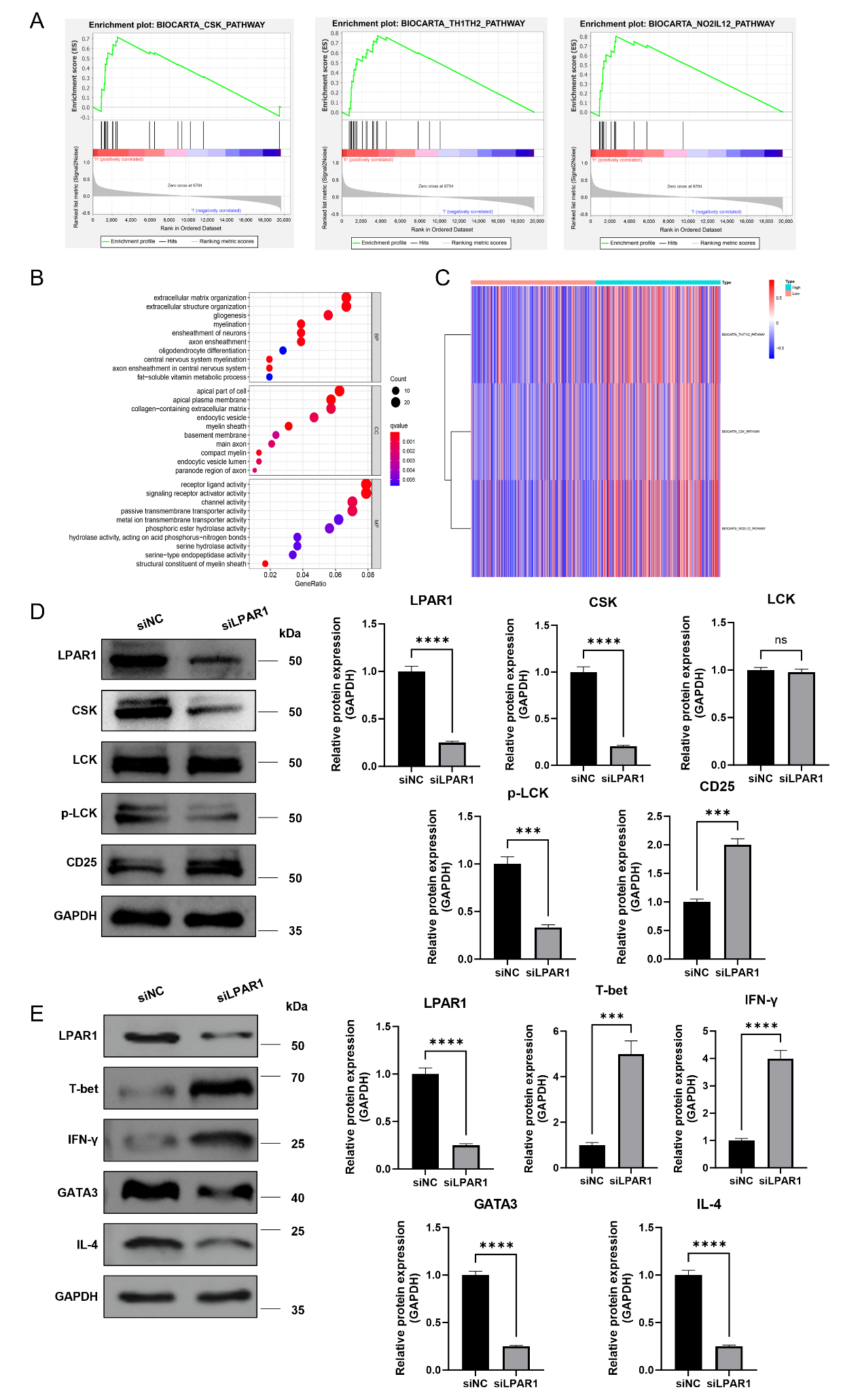  **siLPAR1**  **siNC**  **LPAR1 50 kDa** |
| --- |
| Extended Data Figure 7. The original blotting of LPAR1 in Figure 5E. Left, original blotting of LPAR1 and corresponding sample names; Right, cropped version in manuscript. |
| 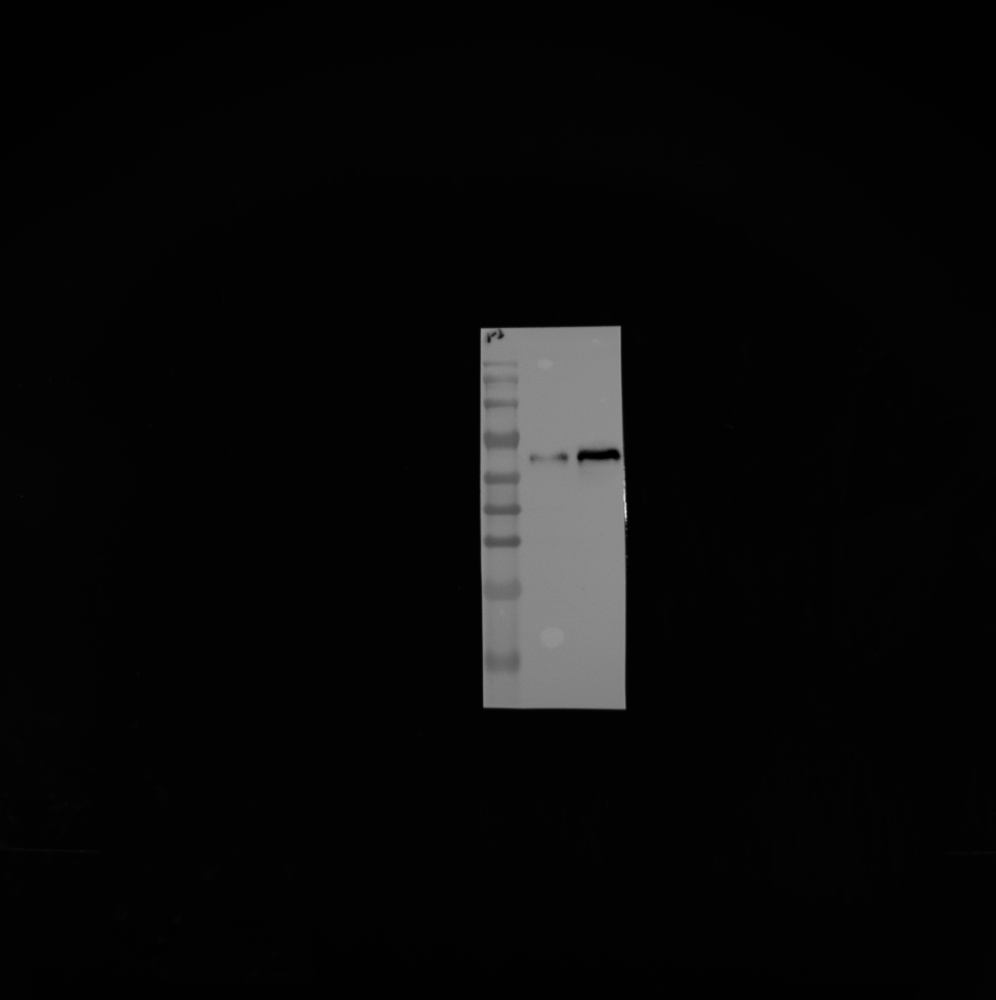 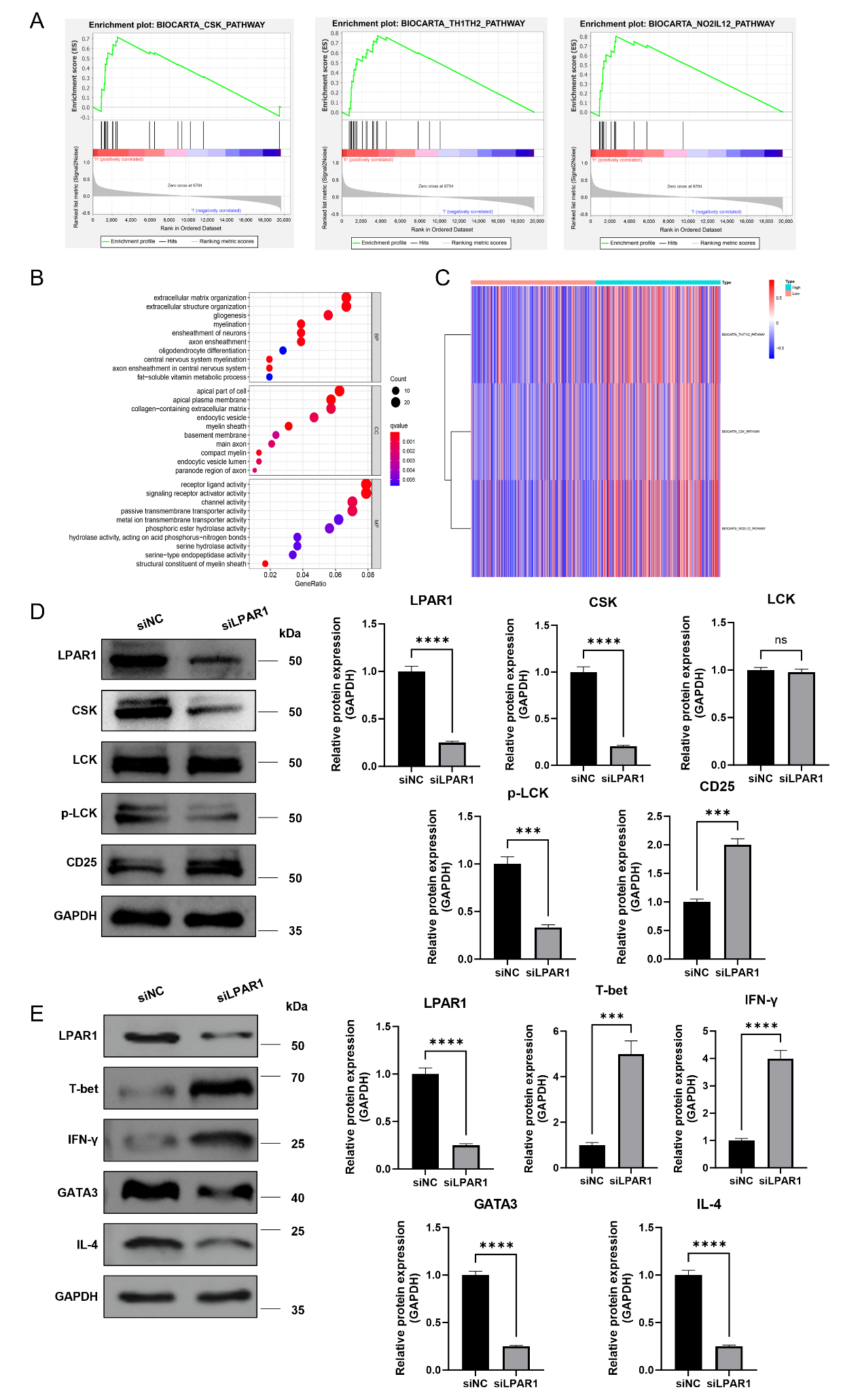  **siLPAR1**  **T-bet 62 kDa**  **siNC** |
| Extended Data Figure 8. The original blotting of T-bet in Figure 5E. Left, original blotting of T-bet and corresponding sample names; Right, cropped version in manuscript |
| 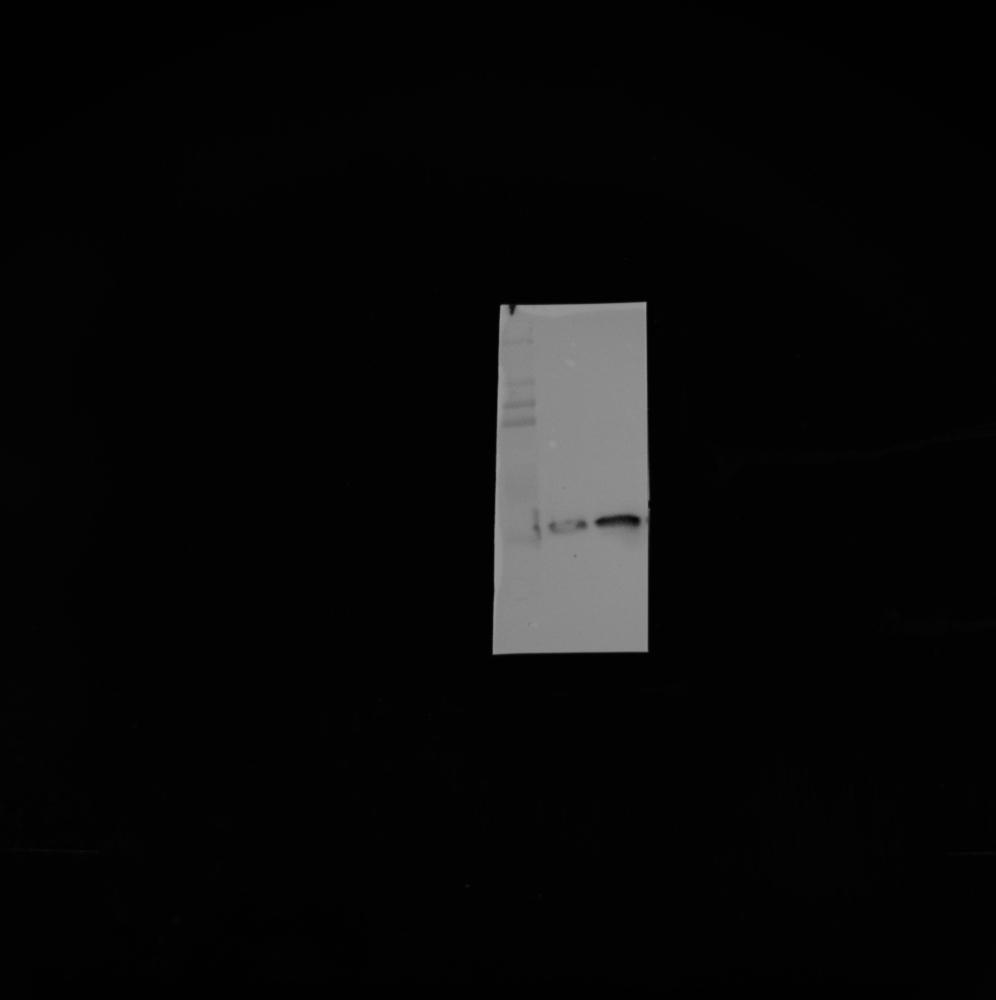 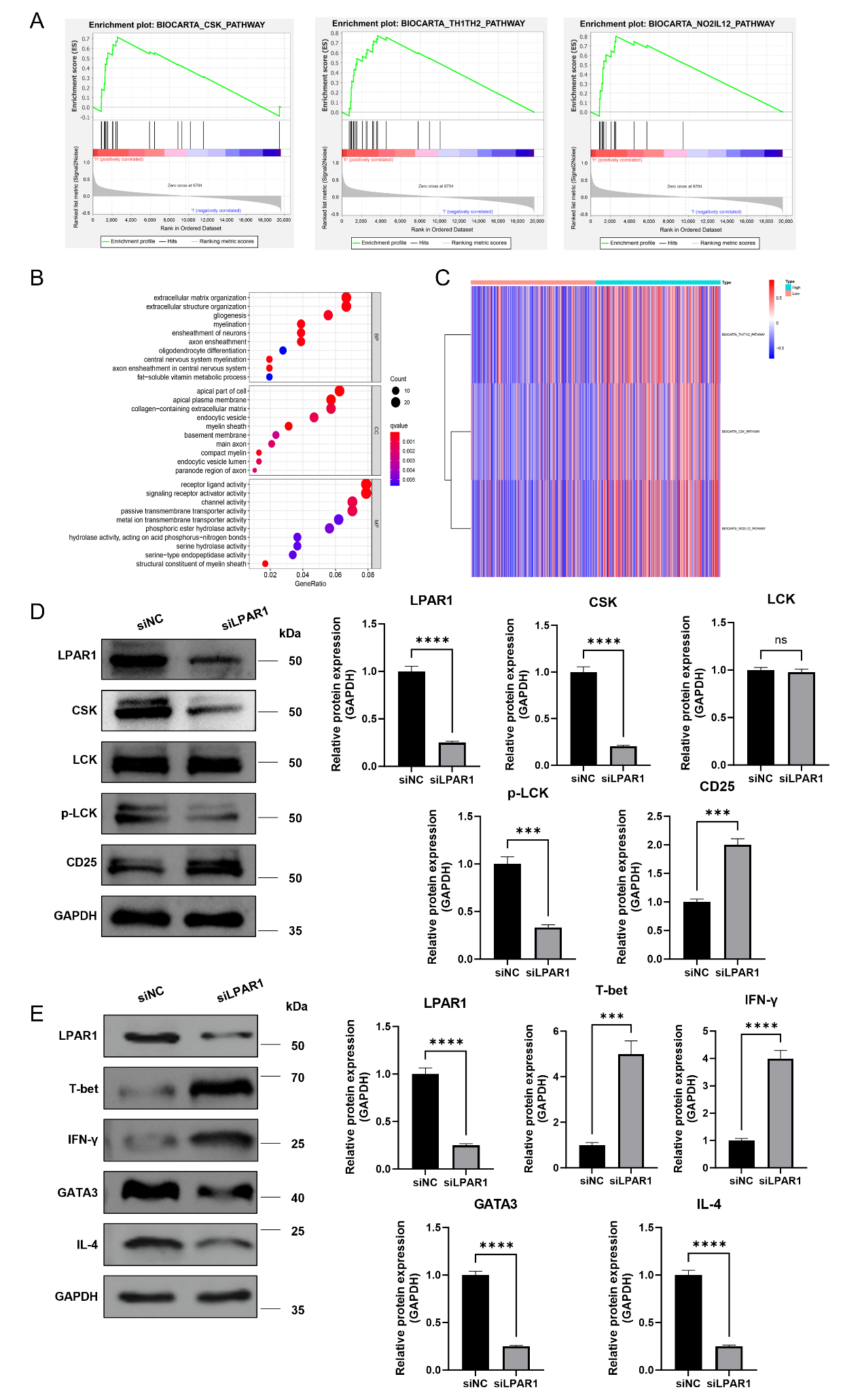  **siLPAR1**  **IFN-γ 22 kDa**  **siNC** |
| Extended Data Figure 9. The original blotting of IFN-γ in Figure 5E. Left, original blotting of IFN-γ and corresponding sample names; Right, cropped version in manuscript |
| 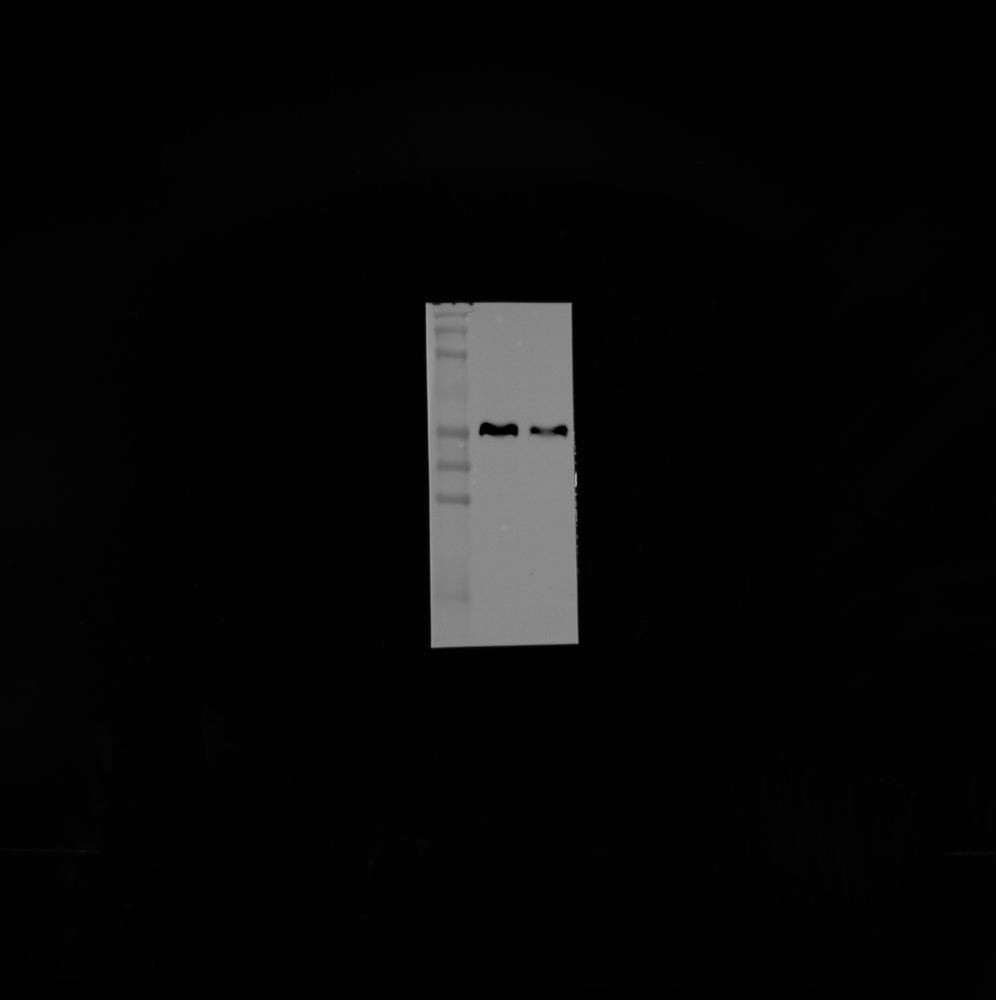 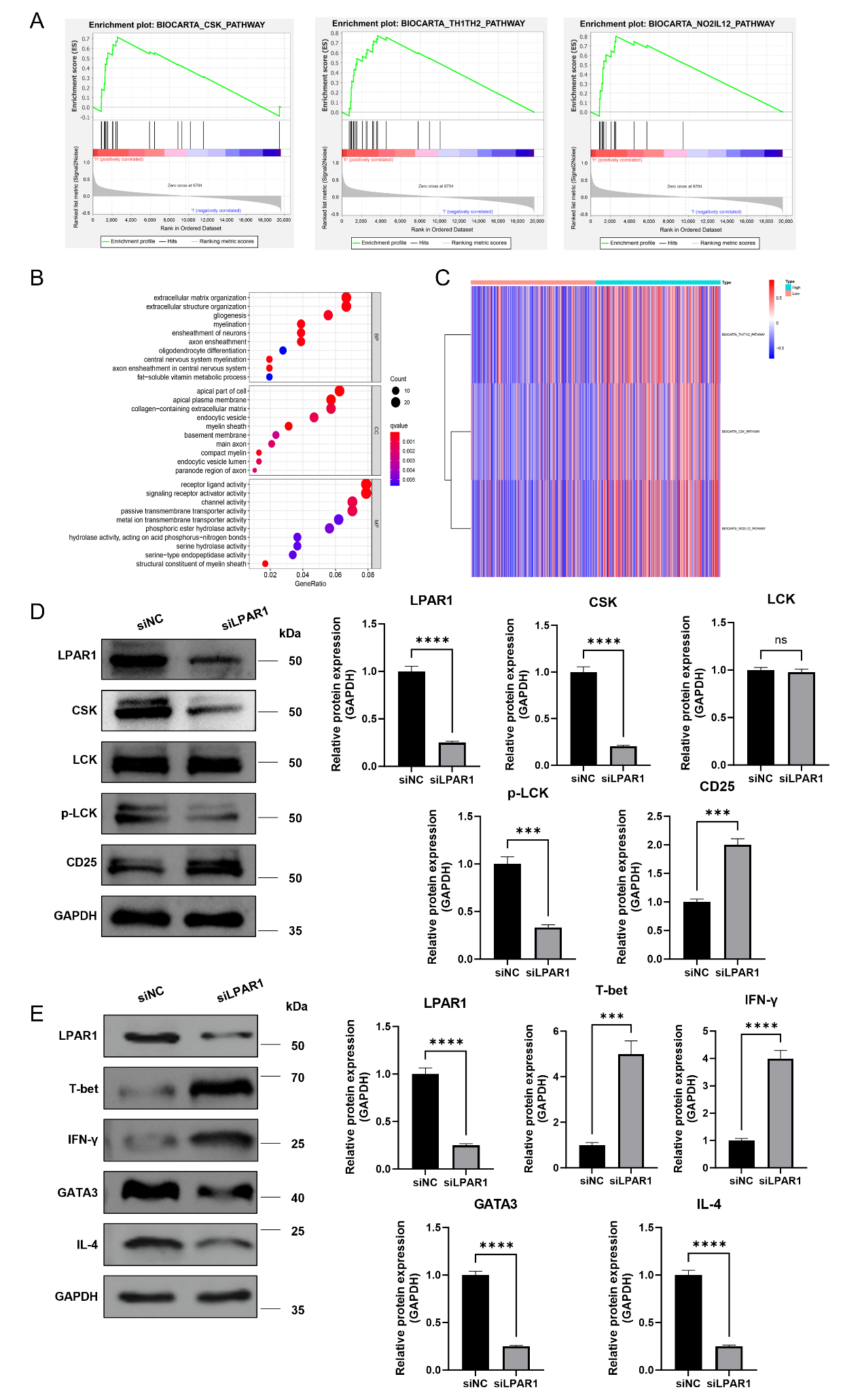  **siLPAR1**  **siNC**  **GATA3 48 kDa** |
| Extended Data Figure 10. The original blotting of GATA3 in Figure 5E. Left, original blotting of GATA3 and corresponding sample names; Right, cropped version in manuscript |
| 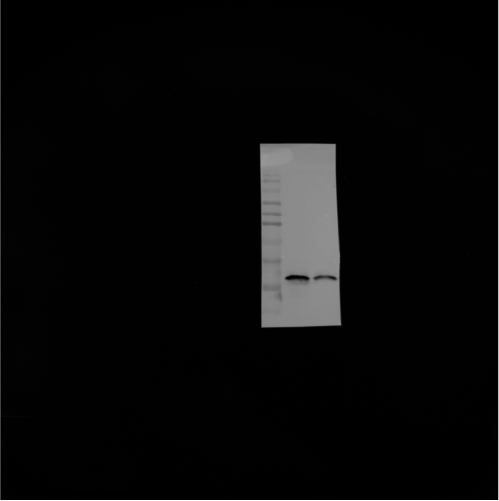 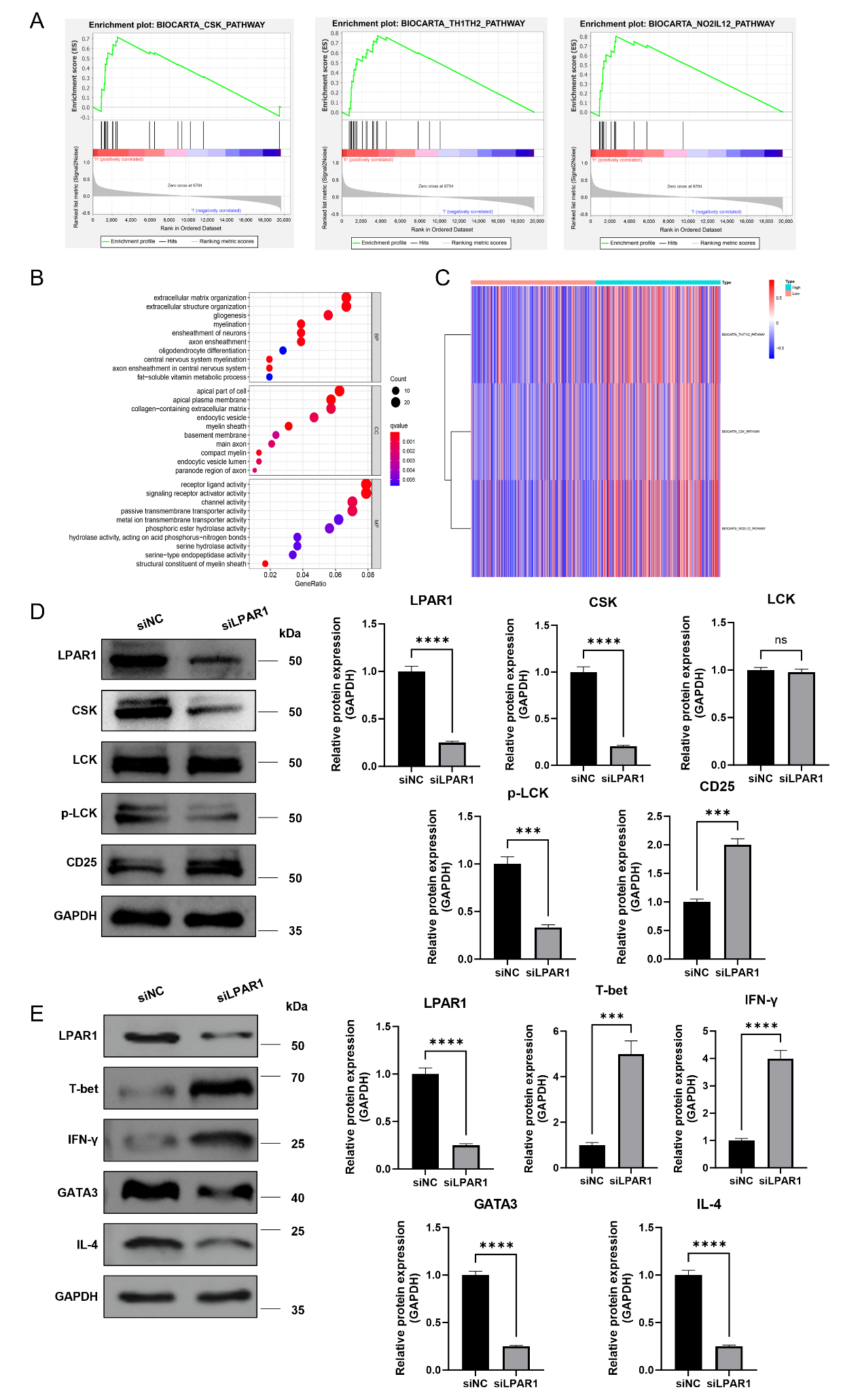  **siNC**  **siLPAR1**  **IL-4 18 kDa** |
| Extended Data Figure 11. The original blotting of IL-4 in Figure 5E. Left, original blotting of IL-4 and corresponding sample names; Right, cropped version in manuscript |
| 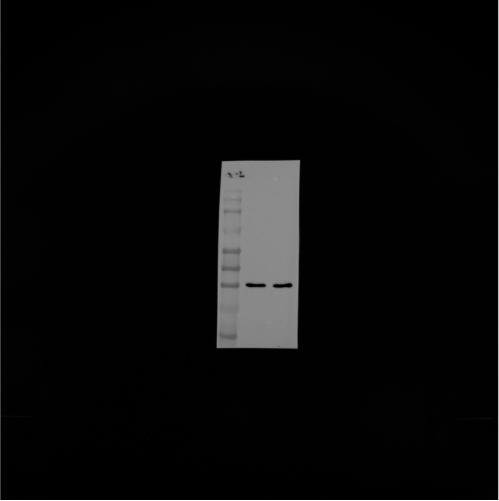 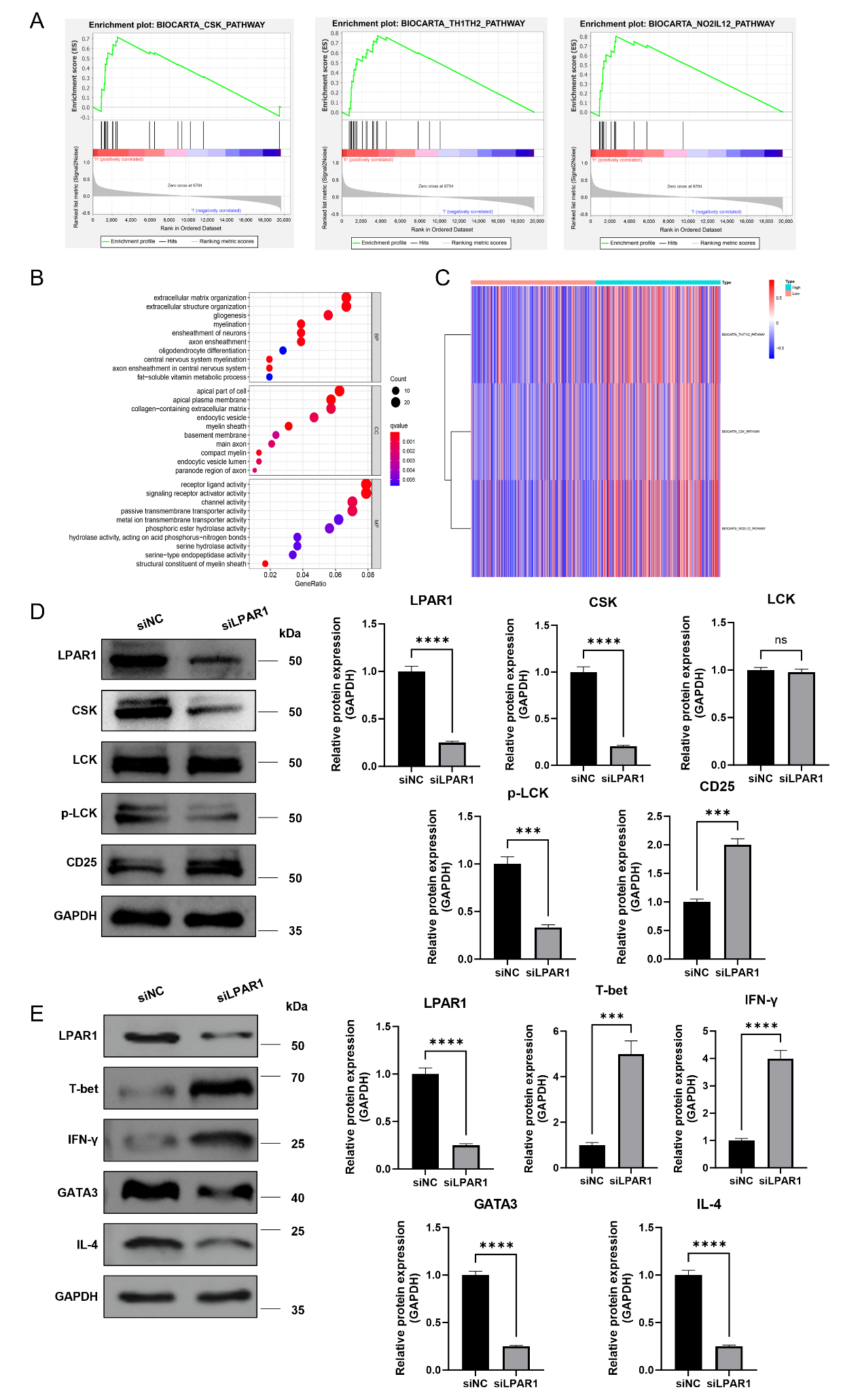  **siNC**  **siLPAR1**  **GAPDH 36 kDa** |
| Extended Data Figure 12. The original blotting of GAPDH in Figure 5E. Left, original blotting of GAPDH and corresponding sample names; Right, cropped version in manuscript |
